# Lay health worker intervention in pre-diabetes management: Study protocol of a pragmatic randomized controlled trial for Chinese families living in inadequate houses

**DOI:** 10.3389/fpubh.2022.957754

**Published:** 2022-10-10

**Authors:** Crystal Ying Chan, Becky Pek-kei Hoi, Eliza Lai-yi Wong

**Affiliations:** ^1^JC School of Public Health and Primary Care, The Chinese University of Hong Kong, Hong Kong, Hong Kong SAR, China; ^2^Center for Health Systems and Policy Research, JC School of Public Health and Primary Care, The Chinese University of Hong Kong, Hong Kong, Hong Kong SAR, China

**Keywords:** pre-diabetes, primary care, task-shifting, protocol, disease management, community-based participatory research, community participation, community health worker

## Abstract

**Introduction:**

Lay health workers, despite their lack of formal trainings, are important partners in providing accessible care to people with risk to develop diabetes in the community. While pre-diabetes and diabetes are more prevalent among people with low socio-economic status, including those living in inadequate houses. However, this population often have accessibility problems to formal care services due to their financial and social disadvantages. In a high-income, developed Chinese society, this pragmatic randomized controlled trial seeks to investigate the effect of a 6-months lay health worker intervention in diabetes management among people living in sub-divided flats units in Hong Kong.

**Methods and analysis:**

In this trial, 222 Chinese primary caregivers living in inadequate houses and with diabetes risk will be recruited *via* non-profit organizations serving in districts with low average household incomes and prevalent subdivided flats in Hong Kong. Adopting a 6 months wait-list control, participants will be randomized to receive a 6-months lay health worker intervention of 5 components, including (1) lay health worker training and support; (2) health professional training; (3) formulation of a targeted care plan for the health and nutritional needs of the families; (4) case management approach; and (5) financial subsidy for lay health workers to sustain the practice. The control group will receive usual care and health information on diabetes risk management. Glycated hemoglobin (HbA1c) and fasting blood glucose will be taken at the entry and exit assessment of this trial as primary outcomes.

**Discussion:**

Our randomized controlled trial is one of the first to investigate the effect of lay health worker intervention on pre-diabetes management in a low-income Chinese population residing in inadequate houses. This study could provide insights to consider alternative service provision models to people living with diabetes risk in the community, by providing a care option to be supported by community health workers and enhanced community participation of care providers. This study attempts to evaluate the impact of a lay health worker intervention using a mixed-method study design. Despite its contribution, this study might be subjected to sampling bias since all the participants will be recruited from non-profit organizations serving deprived populations.

**Trial registration number:**

ChiCTR2100052080 in Chinese Clinical Trial Registry. URL: https://www.chictr.org.cn/edit.aspx?pid=134928&htm=4.

## Introduction

Diabetes is a major cause of preventable deaths globally. In 2019, diabetes affected 463 million populations and caused 1.5 million extra deaths directly (1). The developed, high-income countries have the highest prevalence, with 10% of the population is diagnosed with diabetes (2). Pre-diabetes and diabetes (including impaired fasting glycemia and impaired glucose tolerance, IGT), can be diagnosed by glycated hemoglobin (HbA1C) or 2-h plasma glucose based on 75-gram oral glucose tolerance test (OGTT). Without proper intervention, people with diabetes might be complicated from blindness, kidney failure, heart attacks, stroke, and lower limb amputation, which collectively contributes to 4.2 million deaths globally (2). Given the global cost of pre-diabetes and diabetes, early interventions and cost-effective disease management strategies offer an opportunity to reduce preventable disabilities and all-cause mortality in the general population.

Among all populations, people with lower socio-economic status are more prone to be identified with pre-diabetes/diabetes especially in developed economies. Socio-economic status is a multidimensional concept generally refers to the individual's income level, employment status, and educational attainment (3, 4). In the United States, the prevalence of diabetes was inversely related to household poverty level, which the group in the lowest federal poverty level has twice the odds of having diabetes compared to those ranked the highest (5). It can be explained by the findings of a meta-analysis, which indicates that low income, low educational level and low occupation were associated with a 40, 45, and 31% increased risk of developing type 2 diabetes in high-income countries (6). Similarly, in developed countries, pre-diabetes is more prevalent in the population with lower socio-economic conditions (7). On the other hand, low socio-economic status also impairs the management of diabetes. There is considerable evidence that low socio-economic status increases the risk of diabetes-related complications (8) and mortality (9).

Poor socio-economic status could limit individuals' choices of the living environment, and eventually lead to residing in inadequate houses. According to the United Nations, inadequate housing is a residence which fails any of the following criteria: (1) security of tenure; (2) availability of services, materials, facilities and infrastructure; (3) affordability; (4) habitability; (5) accessibility, (6) location, or (7) cultural adequacy (10). Deprived households might not be able to afford whole-flat residences, and eventually live in sub-divided flats. In Hong Kong, a developed high-income region in China, there are 0.2 million population residing in sub-divided flats in Hong Kong in 2016 (11). A survey revealed that more than 90% of the sub-divided flats households failed to meet at least one criterion of adequate housing, while half of the respondents did not have an independent kitchen and suffer from pest problems (12, 13). Sharing the challenge of worsening inequities with other Asian developed cities, Hong Kong have a Gini co-efficient of 0.54 in 2016, and its average flat price has rocket-raised to the top globally, indicating a worsening income inequality and difficulties in home ownership of the citizen (11). Against the backdrop of its extreme income disparity and growth of SDU resident size, Hong Kong could be an example of a high-income, developed city to investigate the health needs of people in inadequate environments in Asia.

Lay health workers are important partners in managing chronic disease including diabetes, through performing tasks related to health care delivery, despite their lack of formal professional trainings (14). Their responsibilities include facilitating the adoption of healthy practices, promoting access to care, supporting primary and chronic care, and advocating structural changes to cater to the health needs of a community (15). Lay health workers are particularly suitable for delivering support to hard-to-reach populations at a relatively low cost. Residing in the same community, lay health workers have shared backgrounds and connections with the people they are serving and therefore could provide assistance that is more culturally-sensitive and appropriate (14, 16). A randomized control trial has resulted in a reduction in HbA1c levels, body mass index and waist circumference of diabetic patients in 12 months. The diabetic patients in the intervention group have received three courses delivered by trained lay health workers with diabetes every 3 months, while the control group has received usual care (17). Another randomized control trial with a lay health worker intervention has also significantly reduced the HbA1c levels in diabetic patients. Patients were assigned to a control group with usual care, and an intervention group with trained lay health workers working with a registered nurse. Over the 12 months, the lay health workers have provided continuous support to the patients by conducting health education sessions, solving problems in diabetic self-management, mobilizing family support, and reinforcing adherence to medical appointment and medication regimens. Meanwhile, the nurse arranged meeting with patients, provided feedback to the physicians and supervised the work of the lay health workers (18).

However, there are inconclusive conclusion of the intervention effectiveness and further study is needed. There were three systematic reviews conducted on the effectiveness of lay health worker interventions on diabetes management (19–21). Authors note that there was high variability in study designs, diseases outcomes and measurements, leading to high risk of bias in the current studies (19–21). Also, some studies on lay health intervention of diabetes management did not clearly differentiate the intervention for people with type 1 and type 2 diabetes, and did not mention details of trainings provided to the lay health workers. There were also a wide range of duties covered by lay health worker in these studies, including language interpretation, conducting educational sessions, and providing health assessment (19–21). There was a high heterogeneity in content, duration, frequency and intensity of existing lay health worker intervention in literature and a robustly designed evaluation is needed to draw conclusion on its effectiveness.

This is a pragmatic, wait-list randomized controlled trial to evaluate the impact of lay health worker intervention on the incidence and disease management of pre-diabetes among people living in inadequate households. We hypotheses that the lay health worker intervention could significantly improve disease prognosis of people with a risk to develop diabetes, who are living in inadequate housing using an example of Hong Kong Chinese population.

## Methods and analysis

### Study design

**Figure 1:**
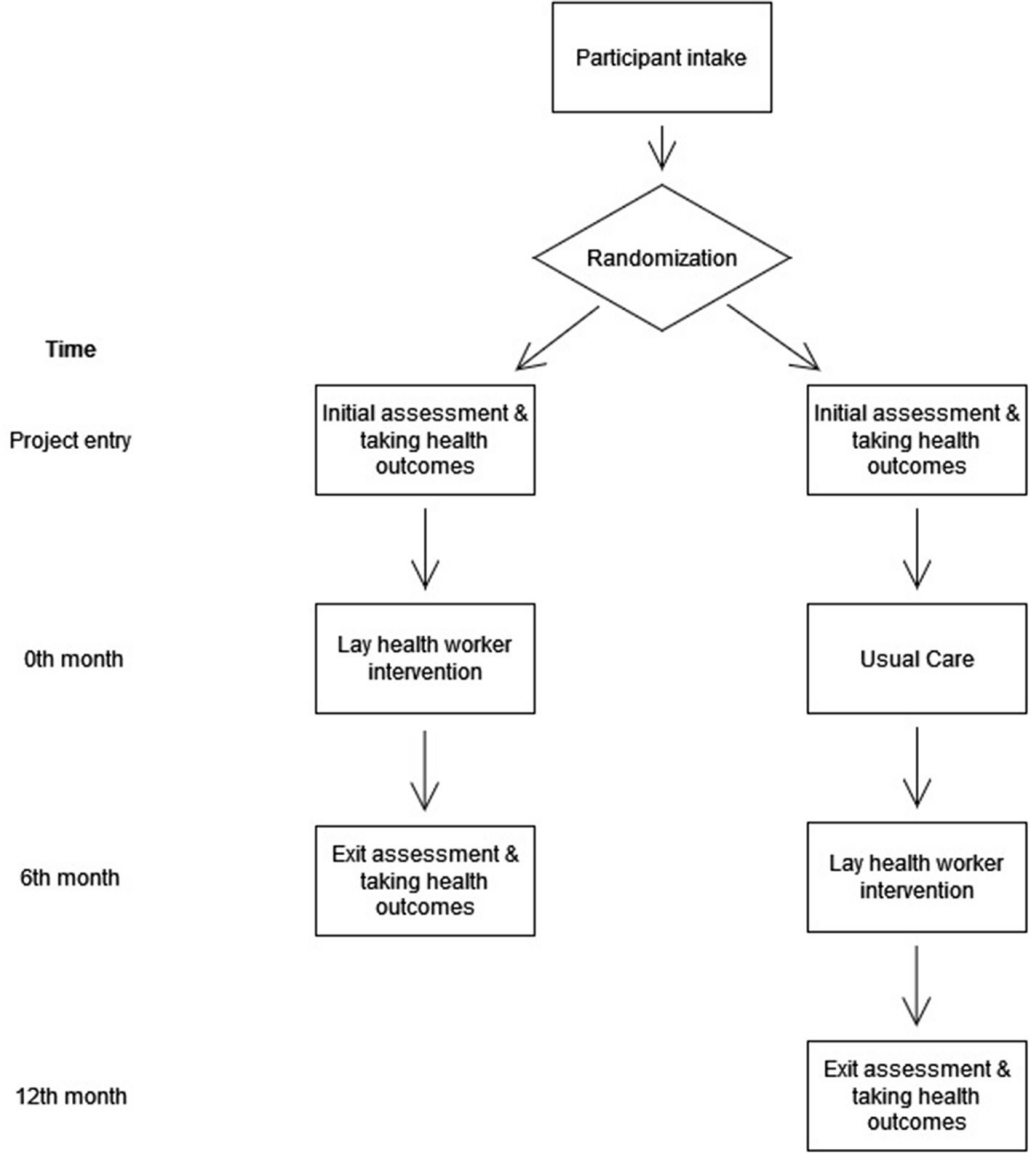
CONSORT workflow of this trial.

This randomized controlled trial aims to evaluate the impact of a lay-health worker intervention on the disease management of type II diabetes mellitus (T2DM) among Chinese sub-divided flat residents units in Hong Kong, comparing to the wait-list controls who receive usual care. This trial is a part of a community, diabetes-screening programme which targets families living in sub-divided homes, aiming to provide disease management to the population diagnosed with diabetes or identified with risk to develop diabetes. This lay worker intervention contains five features, including: (1) lay health worker training and support; (2) health professional training, (3) formulation of a targeted care plan for the health and nutritional needs of the families; (4) case management approach; and (5) financial subsidy for lay health workers to sustain the practice. This trial will be reported in accordance with SPIRIT 2013 checklist (22), as enclosed in Supplementary material S1. Figure 1 comprises the workflow of this trial. Table 1 includes the minimal data set for trial registration.

**Table 1:** Minimal data set for trial registration.

**Data category**	**Information**
Primary registry and trial identifying number	Chinese clinical trial registry ChiCTR2100052080
Date of registration in primary registry	16-Oct-21
Secondary identifying numbers	N/A
Source (s) of monetary or material support	The Hong Kong jockey club charities trust
Primary sponsor	The Hong Kong jockey club charities trust
Secondary sponsor (s)	N/A
Contact for public queries	Crystal Chan, PhD DFPH Jockey club school of public health and primary care, The Chinese University of Hong Kong, China
Contact for scientific queries	Crystal Chan, PhD DFPH Jockey club school of public health and primary care, The Chinese University of Hong Kong, China
Public title	Lay health worker as an example of medical task-shifting: design of a pragmatic randomized controlled trial for diabetes prevention in people living in inadequate housing
Scientific title	Lay health worker intervention on diabetes management
Countries of recruitment	Hong Kong, China
Health condition (s) or problem (s) studied	Diabetes
Intervention (s)	Active comparator: lay health worker intervention
	Placebo comparator: usual care with diabetes control information
Key inclusion and exclusion criteria	Ages eligible for study: ≥ 18 years and < 65 years
	Sexes eligible for study: both
	Accepts healthy volunteers: no
	Inclusion criteria: adult patient (≥ 18 years and < 65 years) who is the major caregiver of households living in sub-divided flat units in Hong Kong
	Exclusion criteria: mentally incapable to give consent
Study type	Interventional
	Allocation: randomized intervention model
	Parallel assignment
	Wait-list control of 6 months waiting period
	Primary purpose: prevention
	Phase I
Date of first enrolment	Nov-21
Target sample size	222
Recruitment status	Recruiting
Primary outcome (s)	Glycated hemoglobin (HbA1c)
Key secondary outcomes	Body mass index; Waist circumference; Percentage body fat; 3-day food records; Depression, anxiety and stress as measured by DASS-21; Quality of life as measured by ED-5D-5L

### Selection of subjects

In this study, participants will be invited by corresponding non-profit organizations (NPOs) in the Kwai Tsing and Kowloon City districts, which are the third district with the lowest average household income, and the third district with the highest percentage of households living in sub-divided units in Hong Kong (11). In 2016, there are in total 12,770 families living in sub-divided units in these two districts, accounting for 13.7% of the total population residing in sub-divided flats in Hong Kong (11).

This trial includes the major caregiver of households living in sub-divided flat units who are (1) responsible for the food preparation of the family, (2) ethnically Chinese and aged ≤ 18 and < 65, (3) identified with ≤ 1 risk factor or symptoms for T2DM according to the *Hong Kong Reference Framework for Diabetes Care for Adults in Primary Care Settings* (19); (4) Hong Kong Identity Document holders, (5) able to communicate in Chinese or English; and (6) able to give consent. People who are cognitively incompetent, and whose co-living family members have already enrolled in this trial will be excluded. *Hong Kong Reference Framework for Diabetes Care for Adults in Primary Care Settings* is a well-established guideline adopted in the primary care services in Hong Kong for screening people who are at risk of T2DM (23). Major caregivers of household are the target and the service recipients of our lay health worker intervention as they are more prone to adverse health outcomes and poorer quality of life due to the physical and mental burden of caregiving duties (24, 25). Also, family caregivers are usually responsible for diet preparation and cooking in the household, and therefore their behavioral change might also enable a change in dietary behavior of the household members.

Eligible participants will be invited to participate in this study through partnering NPOs that provided care services to low-income families living in inadequate housings in Hong Kong. Participants who are interested in the study will be invited to an initial health assessment, where a study information sheet and the ethics consent form will be explained to the participants in person by the research assistant, and participants will be allowed to consider their participation before offering consent. People who are mentally incapable to offer consent will be excluded. The consent form to be used is enclosed in Supplementary material S2.

We will recruit participants from four NPOs in the Kwai Tsing, and Kowloon City districts in Hong Kong to participate in this study. In Hong Kong, social care services are delivered by NPOs and a list of NPOs subverted by public funding was publicly accessible at the Social Welfare Department, which multiple NPOs are responsible for the service delivery in each district. In this study, we do field investigation in the initial design stage of this protocol, and identify NPOs that are responsible for low-income family's services in the districts. The initial communication was established between the research team and the NPOs, of which four of them have agreed to be our recruitment sites for this project.

### Recruitment and training of lay health workers

Lay health workers will be recruited from retired population, local tertiary education institutes, and from the local community through collaboration with NGOs. Potential lay health worker who are (1) either fluent in Cantonese, Mandarin or English; (2) accomplish an education of secondary form 3 or above; and (3) committee to attend training session will be invited to an interview. There will be no restriction of age and sex of the lay health worker to be participated in this study. Upon recruitment, every lay health worker will need to go through a 10-h training session, which consists of the knowledge of conducting health assessment, motivational interviewing, and referral to formal support if necessary. The detailed training content and time distribution is presented in Supplementary materials S3 and S4.

Training will be conducted in the way of interactive training workshops given by a nurse, a dietitian, and public health practitioners to provide the introduction of medication management, nutritional knowledge, and health promotion skills. In the training session, the lay health workers will be given a set of training manuals and resources to guide their involvement in the programme. The training manual will be developed with reference to the evidence from the literature review for other volunteer-based training programmes, such as the Get Healthy Information and Coaching Service provided by the New South Wales Government of Australia (26). The training manual will include roles and responsibilities clarification, training in communication skills, needs assessment skills, trust-building advice, health and safety in a home setting, coping strategies for handling unexpected events, and skills to conduct motivational interviewing. Additional resources such as the instruments to be adopted in needs assessment and continuous monitoring of the health of the families, the detailed workflow of carrying the assessments, knowledge on providing basic consultation and identifying life-threatening symptoms, and skills for infection control during the pandemic will be provided. Furthermore, stimulations on the health need assessment, reinforcing behavioral change, and health monitoring will be done in the workshops. This training workshop will be a co-creation of the dietitian, nurse, social worker and public health practitioner in our inter-disciplinary team, which will create and synergies and knowledge transfer of professional knowledge across dietetics, nursing, public health science and social care.

To ensure the quality of training, all the workshops are conducted in an interactive manner and all the lay health workers must achieve a passing grade in the phone practicum and participate in the debriefing. Each workshop has multiple discussion sessions and quizzes to answer questions and provide opportunities for practice. The quiz is in the form of multiple-choice questions, and instructor will discuss the answers with the lay health worker immediately. The main purpose of the quizzes is to ensure the lay health workers understand the main concept of training, and facilitate in-class discussion with the instructors. All answers could be found in the training materials provided.

### Recruitment and training of healthcare professionals

Trainings will be provided to the healthcare professional to facilitate collaboration with the lay health workers. The primary care workers for the participants in community settings, including nurse and dietitians, will be invited to participate a 1.5-h interactive workshops and training in supporting them to familiarize with the role and strengths of the lay health workers. The interactive workshop will be given to them by the dietitian service lead and the nursing service lead in our project team, covering the project introduction, the overview of the service flow and the collaboration model with the lay health workers, and go through the administrative documents that would be used in the service. The health professionals will also receive a protocol to brief them on the rationales and operational details of this trial, and illustrate the clinical workflow of a lay-health worker intervention with real-case scenarios. Since the lay health workers will be responsible for a 6-month follow-up of the participants, the health professionals will learn how to hand over the case properly to the lay health workers. Moreover, the health professionals should expect to receive referrals for further investigation from the lay health workers when any red-flag symptoms or indicators of care plan occurred. During the face-to-face training session, healthcare workers will have a chance to meet their corresponding lay health workers to build rapport and trust in the working relationship, and therefore to facilitate the necessary referral that might happen during the intervention.

Each healthcare profession will be offered a shadowing session to shadow and observe a real-case practice with lay health worker as demonstrated by our project leads. Then, professional staff trainees will participate in a real-case trial session to conduct the intervention themselves with the LHW, and receive spontaneous feedback from our clinical service leads to ensure homogeneity of the service given. The clinical leads in our project team will review the case notes and collect feedbacks from the professional staffs engaging in the intervention regularly to audit the service quality. In both LHW and professional trainings, repeated rounds of real-case practices will be provided if the intervention quality is deemed as unsatisfactory.

### Lay health worker intervention content

A tailored behavioral change plan will be co-formulated by the lay health workers and the healthcare professional to cater the health needs of the participants. Upon the collection of consent, a needs assessment will be conducted to understand the health and nutritional needs of the participants in the community settings. Then, the primary care workers (who is a nurse or dietitian in this trial) will provide a face-to-face initial consultation session with the trial participants, and identify tailored health goals corresponding to the needs of the participants. Toward the end of the initial consultation, nurse/dietitian will introduce lay health workers to the participants, provide necessary information to ensure smooth handover and handling, and facilitate the rapport building between lay health workers and the participants. Then, the lay health workers will formulate a tailored behavioral change plan according to the health goals set, discuss the feasibility and readiness of the plan with participants, and be responsible for the monitoring of the behavioral change, and managing the self-reported health status of the participants since then through telephone consultation, once 2–4-week for 6 months.

This interventional will adopt a case-management approach. Upon the commencement of the tailored behavioral change plan, lay health workers will perform behavioral change monitoring through the 6-months period, under the supervision of the nurse/dietitian. The lay health workers will adopt a case-management approach, which each participant will be follow-up by the same lay health workers throughout the intervention, to allow the change monitoring to be done based on a trusted relationship. Each of the lay health workers will not be managing more than four families in the same period. To better facilitate behavioral change, the lay health workers will adopt the Stages-of-Change Model, which was developed by James Prochaska and Carlo DiClemente in 1983 (27). The model describes personal behavioral change in five stages, namely pre-contemplation, contemplation, preparation, action, maintenance, and relapse. Stage-of-Change Model is widely adopted in health psychology field and behavioral interventions (28, 29). In our proposed intervention, lay health workers will assess which stage the client is in, and motivate the client to move on to the next stage until the maintenance stage (27). During each follow-up, the lay health workers will review pre-established health goals with the participants, identify the facilitators and barriers of the participants' behavioral changes, and provide recommendations for the participants to overcome the identified barriers through motivational interviewing. Motivational interviewing is a “collaborative, goal-oriented style of communication with particular attention to the language of change,” with the aim to strengthen the person's motivation and commitment for the health behavior goal in an accepting and compassionate atmosphere (30).

Alongside the case management duties, lay health workers will continuously assess the physical, mental, and nutritional health status of the participants through patient-reported outcomes. Lay health workers will maintain a close working relationship with the nurse and dietitian. Following each of the telephone follow-up, the lay health workers will participate in a group-based case de-briefing session where they can discuss the difficulties they encountered during the follow-up, and learn from each other's for better consultation skills. Lay health worker will review case progress with nurse/dietitian once every 2–4 weeks, and will discuss the motivators and barriers for the participants' behavioral changes to guide necessary amendments in the care plan. The participants are, therefore, under the co-management of the clinical professional (nurse or dietitian) and the lay health workers. For there is any undesirable outcome identified during the follow-up, the lay health workers will refer the participants for immediate intervention by the nurse and dietitians' management.

Lastly, financial subsidy will be provided to lay health workers to ensure the sustainability of their practice, and as a reward to honor their time spent on providing services to the participants.

### Randomization and binding

This study will adopt wait list control with open labels, as binding is not possible. The sequence of subjects receiving interventions will be determined by randomization after the informed consent is being collected, of each slots of intervention will be separated by 6 months of waiting period. Statisticians independent from the project team will be given randomly generated treatment allocation within sealed, opaque envelops. During the initial health assessment, one of the envelops will be drawn and the participant will be allocated with the drawn sequence after they consent to participate in this trial. During the waiting period, the experimental group of subjects will be allocated to usual care with no intended treatment given.

### Control group selection

This study will have a wait-list control, where the control will receive usual care of health information on diabetes risk management, conventional weight-control management and dietary education during the 6 months waiting period. On completion of the waiting period, the nurse and dietitian will invite the control group's participants for a face-to-face consultation, where the clinical staff and lay health workers will have a chance to identify their service needs and give tailored care plans.

### Primary and secondary outcomes

This controlled trial will use a wide range of clinical and patient-reported outcomes measures to assess the cost-effectiveness of this intervention. The outcomes are set with reference to the *Medical Research Council framework for evaluating complex intervention* (31) and on the *American Diabetes Association's Standards of Medical Care in Diabetes 2021* for diabetes research (32).

We have two primary outcomes in this trial. Glycated hemoglobin (HbA1c) will be measured by self-test kit (Polymer Technology Systems Inc., Whitestown USA), and fasting blood sample for plasma glucose will be measured by blood glucose monitor (ForaCare Inc, Moorpark USA).

We also included anthropometric measures, self-reported outcomes, dietary consumption patterns, and patients' satisfaction of the lay health worker intervention as secondary outcomes. Body mass index will be calculated with body weight (kg) and body height (m) measured, and waist circumference of the participants will be measured to the nearest 0.1 cm. We will measure the percentage body fat using bioimpedance analysis with BC240 (Tanita Corp., Tokyo Japan). Participants' risk of depression, anxiety and stress will be measured by The Depression, Anxiety and Stress Scale - 21 Items (DASS-21) (33). Self-reported health related quality of life will be measured using EQ-5D-5L (34). Consumption pattern of the participants as recorded by three-day food records to capture the day-to-day variations of the participants' diet (35). In order to improve the validity of the food record collected, a written guideline will be given to the participants to guide their completion of the dietary records, together with a filled template and size of standard food containers for reference. Firstly, participants are instructed to complete the food record in 2 weekdays and 1 weekend day. Also, they are encouraged to consume a typical diet, record every meal they have consumed, using bowls and other utensils to measure the amount of foods and drinks, record the portion of pre-packaged foods stated on the food labels, record the ways of cooking, record the recipes and take a picture of the meals consumed. Upon the completion of the food record, a registered dietitian and a group of lay health workers trained by the dietitian will call the participants to review the accuracy and completeness of the food record. Visual aids are used including pictures of foods and food models. The complete food record is then input into Food Processor by an experienced research assistant to generate nutritional values of the participant's diet (36). Patients' satisfaction toward the lay health worker intervention will be measured with a 5-points Likert scale in a self-administered questionnaire to be filled by the patients at the exit assessment for the assessment, with a higher score indicating a more satisfied care experience in the intervention.

### Sample size calculation

Two hundred and twenty-two participants are needed (111 for intervention, 111 for control at a 1:1 case to control ratio) to attain 80% power, 95% level of significance and 20% dropout rate in a population with an expected population standard deviation of 1.5, and an allowable difference of 0.01 to detect a change of 0.5% change in HbA1c concentration. We plan to train 70 lay health workers on a 1:4 lay health worker to patient ratio, and assuming a 20% dropout rate.

### Data collection, management and monitoring

There will be at least two time points for data collection of this intervention trial as demonstrated in Figure 1. For each assessment, the personal information of the participants will be collected through an electronic survey form that is directed link to an encrypted, private domain hosted by the Chinese University of Hong Kong. All information that contains the identification of the participants will be stored separately, and link with the main dataset with a unique identifier. The file containing the personal information of the participants and their corresponding identifier will only be accessible to the research team members. All data will be destroyed 5 years upon the completion of results dissemination of this study.

Data monitoring will be done by an epidemiologist that is independent of the sponsor for this trial. An interim analysis will be done upon the competition for the first block of intervention to evaluate the effectiveness, and to manage and report any adverse events or unintended of the interventions. Stopping rule will be employed to terminate this trial stop if there is evidence of futility or severe and serious adverse reactions due to intervention. For participants who discontinue from intervention, their reasons of discontinuation will be collected. Results of effectiveness, adverse events, and unintended outcomes of this intervention will be reported and disseminated through publishing in open-access, peer-reviewed journals.

### Data analysis

We will adopt both qualitative and qualitative approaches to evaluate the impact of lay health worker intervention in pre-diabetes management in this trial.

A quantitative methodology will be adopted to evaluate the intervention effectiveness by performing group-wise comparison of the primary and secondary outcomes in this study. A CONSORT flowchart will be adopted to present the progress and of this trial. We will adopt an intention-to-treat approach in conducting the analysis, with sensitivity analysis to be done with non-ignorable missing data. Descriptive statistics will be produced for primary and secondary outcomes depending on their measurement scale and data distribution to allow meaningful interpretation. Histogram of the data distribution will be plotted to examine whether the data is parametric, or non-parametric. We will produce mean and standard deviation for parametric data, and use median and inter-quarter range for non-parametric data. Number and percentage will be quoted for ordinal and nominal data. Standardized mean differences of each variable will be produced to evaluate the balance in control and intervention groups, with a value larger than 0.1 indicating an imbalance between groups.

In order to compare the effectiveness of intervention between control and the intervention group at the exit assessment of intervention (6^th^ month), we will use repeated-measures Analysis of Variance (ANOVA) for parametric outcomes. We will consider the measures of data at baseline (0^th^ month) and covariates that are imbalanced in control and intervention groups when conducting ANOVA, and the effect size will be estimated by calculating the Cohen's *d* value. For non-parametric variables, the Friedman test will be done, and Kendall's W value will be produced for effect size estimation. For ordinal data, we will use Generalized Estimating Equations to compare groups at each follow-up (0^th^ month, 6^th^ month, and 12^th^ month), allocation group (intervention and wait-list control), time by group interaction, covariates that are imbalance between groups, and important socio-economic variables including sex, age, and education level. We will use an identify link function given a normal distribution of the captioned outcome. Also, Kaplan-Meier curves will be drawn to capture the comparison of diabetes-free survival in the intervention and control group. The level of significance is set at 5% m and *post-hoc* adjustment will be applied for multiple comparisons. Multiple imputations will be used whenever appropriate for missing data. In this analysis, all statistical analyses will be conducted using R and RStudio, and the Cohen's *d* value will be estimated using G^*^Power.

We will use a mixed-method approach to conduct the process evaluation on the participants. Quantifiable, routine data will be collected to evaluate the output of this intervention in corresponding to the five components of this intervention as described in Table 2, following with a *t*-test to compare intra-groups estimates to allow meaningful interpretations at the 6^th^ and 10^th^ month. The results from quantitative study part will provide a description for the overall execution of the intervention, which inform the answer for the following research questions: (1) what outputs were delivered to achieve the five components of the interventions; and (2) to which extend did the different stakeholders (study participants, healthcare worker, and lay health workers) complete/ adhere to the intervention? In complimentary to the quantitate research, the qualitative part of the mix-method study will be conducted in parallel. Two rounds of qualitative semi-structured, auto-taped individual interviews will be conducted at 6^th^ months (the completion of wait list control period) and 12^th^ month (project endpoint), to collect feedbacks from different stakeholders involved in this project. Project staffs (*n* = 2), lay health workers (*n* = 10), healthcare professionals participated (*n* = 5), NPO collaborators (*n* = 5) and study participants (*n* = 10) will be invited to participate in this study. Each individual interview will last for 1–1.5 h, with the following research questions to be answered: (1) what are the feedback and major concerns of the participants of the programme; (2) how to address these concerns in the second cycle of intervention?; (3) what are the facilitators and barriers in the programme delivery; and (4) how to enhance the facilitators or mitigate the barriers?, as guided by the *Consolidated Framework for Implementation Research* (37). Detailed interview questions can be found in Supplementary material S5. All the interviews will be conducted by research staff that is not involved in the delivery of this trial to ensure impartiality.

**Table 2:** Outline of the process evaluation: output indicators.

**Component**	**Output**	**Indicators**	**Source of data**
1. Lay health worker training	More trained workers	Number of training sessions conducted	Administrative data
		Number of total lay health workers trained	Administrative data
	Lay health workers being more confident to lead intervention	Knowledge, attitude and behavior in leading an intervention	Self-administered survey before and after training
2. Health professional training	More health professionals are equipped with the necessary knowledge to co-work with lay health workers	Number of training sessions conducted	Administrative data
		Number of health professionals trained	Administrative data
		Knowledge, attitude and confidence in collaborating with lay health workers	Self-administered survey before and after training
3. Formulation of targeted care plans	Tailored care plan built for participants	Number of health need assessments conducted	Administrative data
		Number of targeted care plans formed	Clinical and service record
		Completion rate of designed health goals	Clinical and service record
4. Case management	Close and tailored case management for participants	Number of families followed-up by lay health workers	Administrative data
		Number of lay health workers follow-up conducted	Administrative data
		Number of biweekly reviews meeting conducted between lay health workers and nurse/ dietitian	Administrative data
		Number of families followed-up by the same lay health workers	Administrative data
5. Financial subsidy for lay health workers	More lay health workers recruited	Number of lay health workers recruited	Administrative data
	Sustainable service model	Proportion of lay health workers that finished the 6-months follow-up	Administrative data

## Discussion

This is the first pragmatic, randomized controlled trial in evaluating the impacts of lay health worker intervention on the pre-diabetes management of Chinese, sub-divided flats residents with T2DM risk in Hong Kong. Our study findings could supplement the literature gap of real-world evidence in evaluating the benefit of lay health worker intervention in diabetes management in a Chinese population living in comprising housing environment. The use of laboratory data to monitor diabetes prognosis could eliminate potential bias due to the self-reporting, while the randomization arrangement could prevent selection bias and accidental bias. We will adopt a wait-list control in this study by administrative procedure, in order to acquire a comparable control at a more reasonable cost, and to ensure equitable access to our intervention of both the intervention and the control groups.

Despite its contribution to the field, there are challenges surrounding the generalizability of the study findings to other populations, and other settings due to its restriction to the population living in compromised housing environments. Our sample population was recruited through NPOs serving deprived households, and therefore sampling bias could occur. To acquire more detailed and generalizable results for the health care provider and decision-makers to establish policy changes, future quantitative and qualitative studies should be done to collect data on health improvement and cost of intervention in a larger, territory-wide sample.

This study is an open label study and therefore blinding is hard to achieve. However, allocation concealment is secured by generating random numbers by drawing lots and keeping the allocation series in a sealed and opaque envelope. Meanwhile, loss to follow-up is minimized by maintaining a good relationship with participants, monthly telephone calls, timely referral to professionals and clinical services, setting up telephone hotline to resolve enquiries, connecting with participants *via* WhatsApp, and providing assistances in times of pandemic.

## Ethics and dissemination

The protocol, informed consent form, procedure to obtain consent, and the procedure to protect confidentiality of personal data of this trial was approved by the Joint Chinese University of Hong Kong – New Territories East Cluster Clinical Research Ethics Committee of the Chinese University of Hong Kong (2021.313), and are registered in the Chinese Clinical Trial Registry (ChiCTR2100052080). Any amendments on the protocol will be communicated though updating in the public webpage of the Trial Registry. The funder has no role in study design; collection, management, analysis, and interpretation of data; writing of the report; and the decision to submit the report for publication. The results of this trial will be published in peer-reviewed journal articles and the final trial dataset will be made available after de-identification of the participants.

To ensure confidentiality of the participants, all information collected from respondents will be locked in a password-protected computer. Personal data (name, identity document, and any other personal identifiable information) will not be recorded on the project's data sheets or electronic files. Instead, each participant will be assigned a unique study identifier. The document or electronic file containing the linkage information between study code and the identity of the participants will be kept separate from the study data files. Database with names will be kept in locked cabinet to which is only accessed by staff involving in this study, and electronic data will be saved in secured computer with restricted access. The authors of this protocol will be responsible for the safekeeping of the personal data involved in this study.

## Conclusion

Lay health worker is a possible solution to lessen medical manpower stress and provide a continuum of care in population with problem to access medical services. This study aims to fill a knowledge gap in finding the best practice for shifting the care load to partners in the community, and rigorously examine the effectiveness of lay health worker intervention in diabetes prevention among a low-income, Chinese population. Research findings could shed lights on future health services delivery, by providing a promising cost-effective solution to delivery diabetes care to the deprived population in developed world.

## Ethics statement

The studies involving human participants were reviewed and approved by the Joint Chinese University of Hong Kong – New Territories East Cluster Clinical Research Ethics Committee of the Chinese University of Hong Kong. Written informed consent to participate in this study was provided by the participants' legal guardian/next of kin.

## Author contributions

CYC, BP-kH, and EL-yW contributed to the design of the protocol. CYC and BP-kH drafted the manuscript. EL-yW conceived the study and were in charge of overall direction and planning. All authors contributed to the article and approved the submitted version.

## Funding

This work was supported by The Hong Kong Jockey Club Charities Trust (Grant Number 2021-0274).

## Conflict of interest

The authors declare that the research was conducted in the absence of any commercial or financial relationships that could be construed as a potential conflict of interest.

## Publisher's note

All claims expressed in this article are solely those of the authors and do not necessarily represent those of their affiliated organizations, or those of the publisher, the editors and the reviewers. Any product that may be evaluated in this article, or claim that may be made by its manufacturer, is not guaranteed or endorsed by the publisher.
