# Supplementary material for: Lay health worker intervention in pre-diabetes management: Study protocol of a pragmatic randomized controlled trial for Chinese families living in inadequate houses
Source: Front Public Health. 2022 Oct 10;10:957754. doi: 10.3389/fpubh.2022.957754 (PMC9589092; doi:10.3389/fpubh.2022.957754)
Supplement: Supplementary file 1 [file Presentation_1.pdf]

Supplementary Material

S1. SPIRIT check list recommended items to address in a clinical trial protocol and related documents

Section/item	ItemNo	Description	Page
**Administrative information**			
Title	1	Descriptive title identifying the study design, population, interventions, and, if applicable, trial acronym	1
trial registration	2a	Trial identifier and registry name. If not yet registered, name of intended registry	1
	2b	All items from the World Health Organization Trial Registration Data Set	4-6
Protocol version	3	Date and version identifier	1
Funding	4	Sources and types of financial, material, and other support	14
Roles and responsibilities	5a	Names, affiliations, and roles of protocol contributors	14
	5b	Name and contact information for the trial sponsor	14
	5c	Role of study sponsor and funders, if any, in study design; collection, management, analysis, and interpretation of data; writing of the report; and the decision to submit the report for publication, including whether they will have ultimate authority over any of these activities	13
	5d	Composition, roles, and responsibilities of the coordinating centre, steering committee, endpoint adjudication committee, data management team, and other individuals or groups overseeing the trial, if applicable (see Item 21a for data monitoring committee)	14
**Introduction**			

Background and rationale	6a	Description of research question and justification for undertaking the trial, including summary of relevant studies (published and unpublished) examining benefits and harms for each intervention	3-4
	6b	Explanation for choice of comparators	4
Objectives	7	Specific objectives or hypotheses	4
Trial design	8	Description of trial design including type of trial (eg , parallel group, crossover, factorial, single group), allocation ratio, and framework (eg , superiority, equivalence, noninferiority, exploratory)	4
**Methods: Participants, interventions, and outcomes**			
Study setting	9	Description of study settings (eg , community clinic, academic hospital) and list of countries where data will be collected. Reference to where list of study sites can be obtained	4-6
Eligibility criteria	10	Inclusion and exclusion criteria for participants. If applicable, eligibility criteria for study centres and individuals who will perform the interventions (eg , surgeons, psychotherapists)	6
Interventions	11a	Interventions for each group with sufficient detail to allow replication, including how and when they will be administered	7-9
	11b	Criteria for discontinuing or modifying allocated interventions for a given trial participant (eg , drug dose change in response to harms, participant request, or improving/worsening disease)	10
	11c	Strategies to improve adherence to intervention protocols, and any procedures for monitoring adherence (eg , drug tablet return, laboratory tests)	7, 9
	11d	Relevant concomitant care and interventions that are permitted or prohibited during the trial	9

Outcomes	12	Primary, secondary, and other outcomes, including the specific measurement variable (eg , systolic blood pressure), analysis metric (eg , change from baseline, final value, time to event), method of aggregation (eg , median, proportion), and time point for each outcome. Explanation of the clinical relevance of chosen efficacy and harm outcomes is strongly recommended	9-10
Participant timeline	13	Time schedule of enrolment, interventions (including any run-ins and washouts), assessments, and visits for participants. A schematic diagram is highly recommended (see Figure)	10
Sample size	14	Estimated number of participants needed to achieve study objectives and how it was determined, including clinical and statistical assumptions supporting any sample size calculations	10
Recruitment	15	Strategies for achieving adequate participant enrolment to reach target sample size	6
**Methods: Assignment of interventions (for controlled trials)**			
Allocation:			6
Sequence generation	16a	Method of generating the allocation sequence (eg , computer-generated random numbers), and list of any factors for stratification. To reduce predictability of a random sequence, details of any planned restriction (eg , blocking) should be provided in a separate document that is unavailable to those who enrol participants or assign interventions	6
Allocation concealment mechanism	16b	Mechanism of implementing the sequence allocation (eg , central telephone; sequentially numbered, opaque, sealed envelopes), describing any steps to conceal the sequence until interventions are assigned	6
Implementation	16c	Who will generate the allocation sequence, who will enrol participants, and who will assign participants to interventions	6

Blinding (masking)	17a	Who will be blinded after assignment to interventions (eg , trial participants, care providers, outcome assessors, data analysts), and how	6
	17b	If blinded, circumstances under which unblinding is permissible, and procedure for revealing a participant's allocated intervention during the trial	6
**Methods: Data collection, management, and analysis**			
Data collection methods	18a	Plans for assessment and collection of outcome, baseline, and other trial data, including any related processes to promote data quality (eg , duplicate measurements, training of assessors) and a description of study instruments (eg , questionnaires, laboratory tests) along with their reliability and validity, if known. Reference to where data collection forms can be found, if not in the protocol	10
	18b	Plans to promote participant retention and complete follow-up, including list of any outcome data to be collected for participants who discontinue or deviate from intervention protocols	10
Data management	19	Plans for data entry, coding, security, and storage, including any related processes to promote data quality (eg , double data entry ; range checks for data values). Reference to where details of data management procedures can be found, if not in the protocol	10-11
Statistical methods	20a	Statistical methods for analysing primary and secondary outcomes. Reference to where other details of the statistical analysis plan can be found, if not in the protocol	10-11
	20b	Methods for any additional analyses (eg , subgroup and adjusted analyses)	10
	20c	Definition of analysis population relating to protocol non-adherence (eg , as randomised analysis), and any statistical methods to handle missing data (eg , multiple imputation)	10-11
**Methods : Monitoring**			

Data monitoring	21a	Composition of data monitoring committee (DMC); summary of its role and reporting structure; statement of whether it is independent from the sponsor and competing interests; and reference to where further details about its charter can be found, if not in the protocol. , an explanation of why a DMC is not needed	10
	21b	Description of any interim analyses and stopping guidelines, including who will have access to these interim results and make the final decision to terminate the trial	10
Harms	twenty two	Plans for collecting, assessing, reporting, and managing solicited and spontaneously reported adverse events and other unintended effects of trial interventions or trial conduct	10
Auditing	twenty three	Frequency and procedures for auditing trial conduct, if any, and whether the process will be independent from investigators and the sponsor	NA: Not a clinical trial
**Ethics and dissemination**			
Research ethics approval	twenty four	Plans for seeking research ethics committee/institutional review board (REC/IRB) approval	13
Protocol amendments	25	Plans for communicating important protocol modifications (eg , changes to eligibility criteria, outcomes, analyses) to relevant parties (eg , investigators, REC/IRBs, trial participants, trial registries, journals, regulators)	13
Consent or assert	26a	Who will obtain informed consent or assert from potential trial participants or authorised surrogates, and how (see Item 32)	6
	26b	Additional consent provisions for collection and use of participant data and biological specimens in ancillary studies, if applicable	NA: No biological specimens collected
Confidentiality	27	How personal information about potential and enrolled participants will be collected, shared, and maintained in order to protect confidentiality before, during, and after the trial	14

Declaration of interests	28	Financial and other competing interests for principal investigators for the overall trial and each study site	14
Access to data	29	Statement of who will have access to the final trial dataset, and disclosure of contractual agreements that limit such access for investigators	13
Ancillary and post-trial care	30	Provisions, if any, for ancillary and post-trial care, and for compensation to those who suffer harm from trial participation	NA: Not a clinical trial
Dissemination policy	31a	Plans for investigators and sponsors to communicate trial results to participants, healthcare professionals, the public, and other relevant groups (eg , via publication, reporting in results databases, or other data sharing arrangements), including any publication restrictions	13
	31b	Authorship eligibility guidelines and any intended use of professional writers	13
	31c	Plans, if any, for granting public access to the full protocol, participant-level dataset, and statistical code	13
**Appendices**			
Informed consent materials	32	Model consent form and other related documentation given to participants and authorised surrogates	S3
Biological specimens	33	Plans for collection, laboratory evaluation, and storage of biological specimens for genetic or molecular analysis in the current trial and for future use in ancillary studies, if applicable	NA: No biological specimen collected

*It is strongly recommended that this checklist be read in conjunction with the SPIRIT 2013 Explanation & Elaboration for important clarification on the items. Amendments to the protocol should be tracked and dated. The SPIRIT checklist is copyrighted by the SPIRIT Group under the Creative Commons“ Attribution- NonCommercial - NoDerivs 3.0 Un ported ” license.

S2. English translation of consent form to be used

The Chinese University of Hong Kong Faculty of Medicine

Jockey Club School of Public Health and Primary Care

Lay Health Worker Intervention in Pre-diabetes Management

Participant Consent Form

Research purposes

Families living in small spaces are more likely to suffer from nutritional and health problems during pandemic. Experience in Australia shows that providing health assistance by phone can effectively improve users' healthy living habits, including controlling chronic diseases, physical inactivity, overweight, and overweight. This service method has not yet been used by subdivided households in Hong Kong. The purpose of this study is to examine the effectiveness of telephone-based health assistance in improving nutrition and health issues in subdivided households . The Jockey Club School of Public Health and Primary Care of The Chinese University of Hong Kong invites you to participate in this research, which will help formulate new long-term care and medical service models for Hong Kong .

Research program

If you agree to participate in this research, you need to sign this consent form to confirm your participation . During the study, you will receive at least two health checkups by a nurse or a dieitian, and you will be randomized to receive telephone health assistance services. The phone health assistance service lasts for six months, and the trained researchers will provide you with up to eight sessions of the phone health assistance service. Before and after the service, our nurses or nutritionists will provide you with a free physical examination and a questionnaire to understand your blood sugar, blood lipids, blood pressure, height-to-weight ratio, body fat ratio, as well as your background, and information on knowledge, attitudes and behaviours related to healthy behaviours. In addition, records related to this study will be kept in a locked drawer in the office of the Faculty Building.

Confidentiality of Information

All information you provide will be kept confidential and used for research purposes only, and your identity will never be disclosed. All your information will be kept strictly confidential. Information obtained in this research may be published or reported, but your name will not be mentioned. Whether you participate or not or the opinions you give will absolutely not affect your future use of all medical services. We may retain and store data collected in the study for a period of 3 years after the study is fully concluded (i.e. the researcher has completed the data analysis for the entire study).

Nature of participation

You do not need to pay for participating in this research. Whether your participation is voluntary, you have the right to refuse to participate, withdraw or refuse to answer any questions, and you will not suffer any penalty or lose your due rights. The researcher also reserves the right to withdraw you from the study if you cannot comply with the requirements of the research procedures or if the researcher deems it appropriate for you to withdraw from the study for any reason .

Enquiry and Contact Method

If you have any questions or enquiries about this research, please contact Dr. Chan Ying, researcher at the Jockey Club School of Public Health and Primary Care, The Chinese University of Hong Kong (Office hours : Monday to Friday, 9:00am to 5:00pm, Tel: 2252 8413) for further understanding. You may also call the CUHK New Territories East Hospital Cluster Joint Clinical Research Ethics Committee on 3505 3935 for questions about the rights of research participants. The Chinese University of Hong Kong New Territories East Hospital Cluster Clinical Research Ethics Joint Committee is one of the authorized bodies to review your records about this research for ethical review purposes.

I have read and understood the contents of this consent form and agree to participate in this research.

____________________	____________________	____________________
Participant Name<br>(Block Letters)	Participant's Signature	Date

____________________	____________________	____________________
Principal Investigator / Researcher Name	Principal Investigator / Researcher Signature	Date

S3. Lay Health Worker Training – Content and Time Distribution

**Content/Hour**	**1**	**2**	**3**	**4**	**5**	**6**	**7**	**8**	**9**	**10**
Health assessment skills	X	X								
Basic Dietetic knowledge			X	X			X	X		
Basic Nursing knowledge			X	X			X	X		
Phone Follow up and Communication skills					X	X				
Phone Follow Up Practicum									X	
Phone Follow Up Practicum Debriefing										X

S4. Example of Training Materials used in Lay Health Worker Training (Basic Nursing knowledge)

INTRODUCTION

Phone FU for LHW

Lay health worker training and practicum

Edwin Chung, RN

Work of lay health worker

1. Case Management
2. Management of chronic illnesses
3. Examples
4. Reviewing video and quizzes (nutritional knowledge and motivational interviewing)
5. Phone FU practicum
6. **Attendance check with voice reply will be done at the beginning, middle and the end of the meeting**

Case Management

1. Most of the cases in this project will be **Hypertension, DM, Obesity and other kind of mental illness**
2. You have to know some basics of such diseases and **identify misconceptions** of our client
3. You have to write down **a summary and make appointment** of phone FU based on both of your availability. Appointment can be made by **using google form**.

Case Management

4. You will have to **enroll in a practicum** in order to ensure that you are competent to conduct phone FU work.

5. Before you enroll in practicum, please **review the videos related to nutritional knowledge and finish the quizzes** provided by the deadline.

Hypertension

1. Definition:
    a. Systolic Blood pressure: 130mmHg
    b. Diastolic Blood pressure: 85 mmHg
2. **Systolic** means pressure in vessels when heart **contracts**
3. **Diastolic** means pressure in vessels when heart **relaxes**

Hypertension

1. Measurement of blood pressure

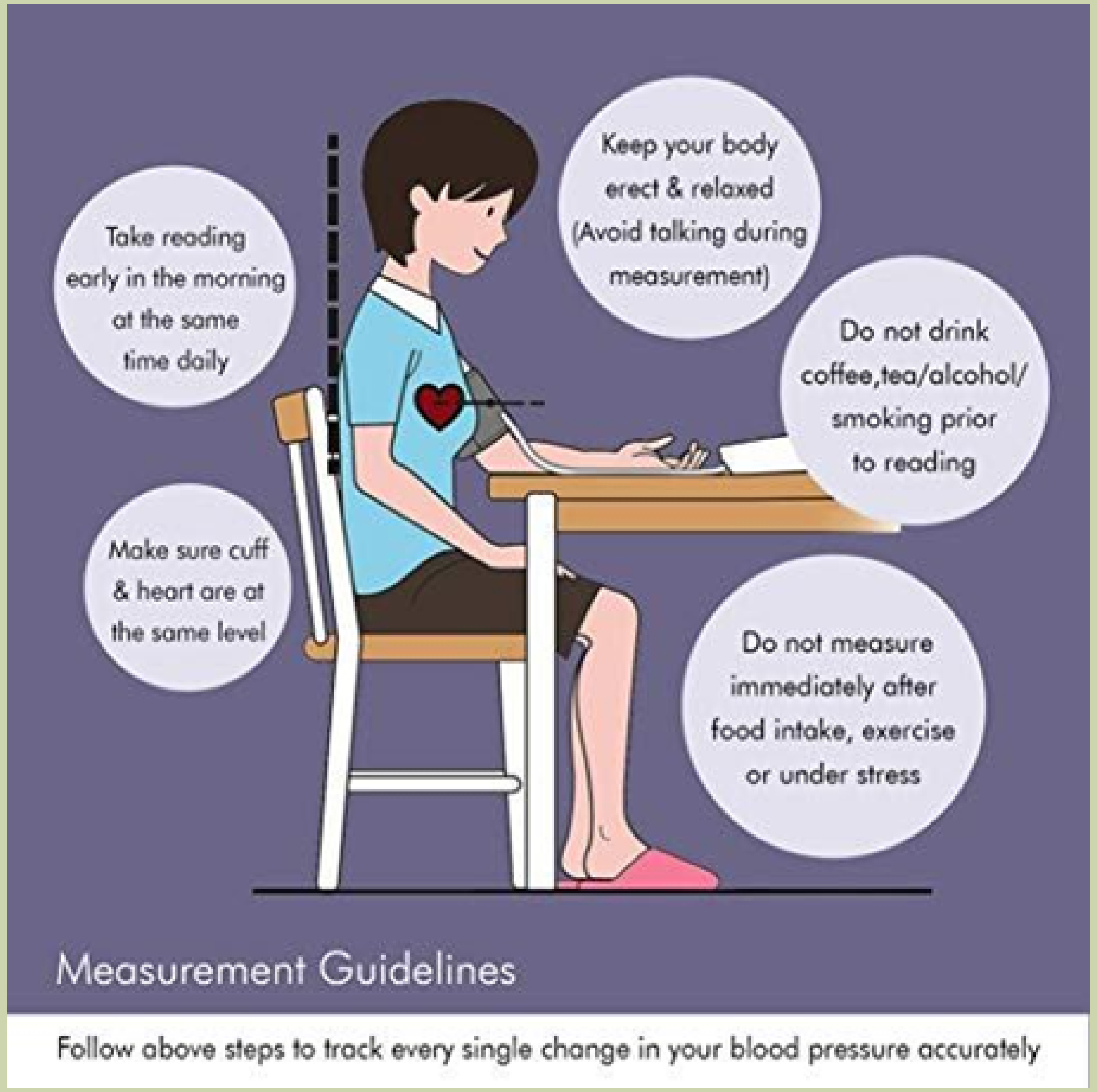

Maintain heart level

Different position will get a different values

Hypertension

1. Measurement of blood pressure

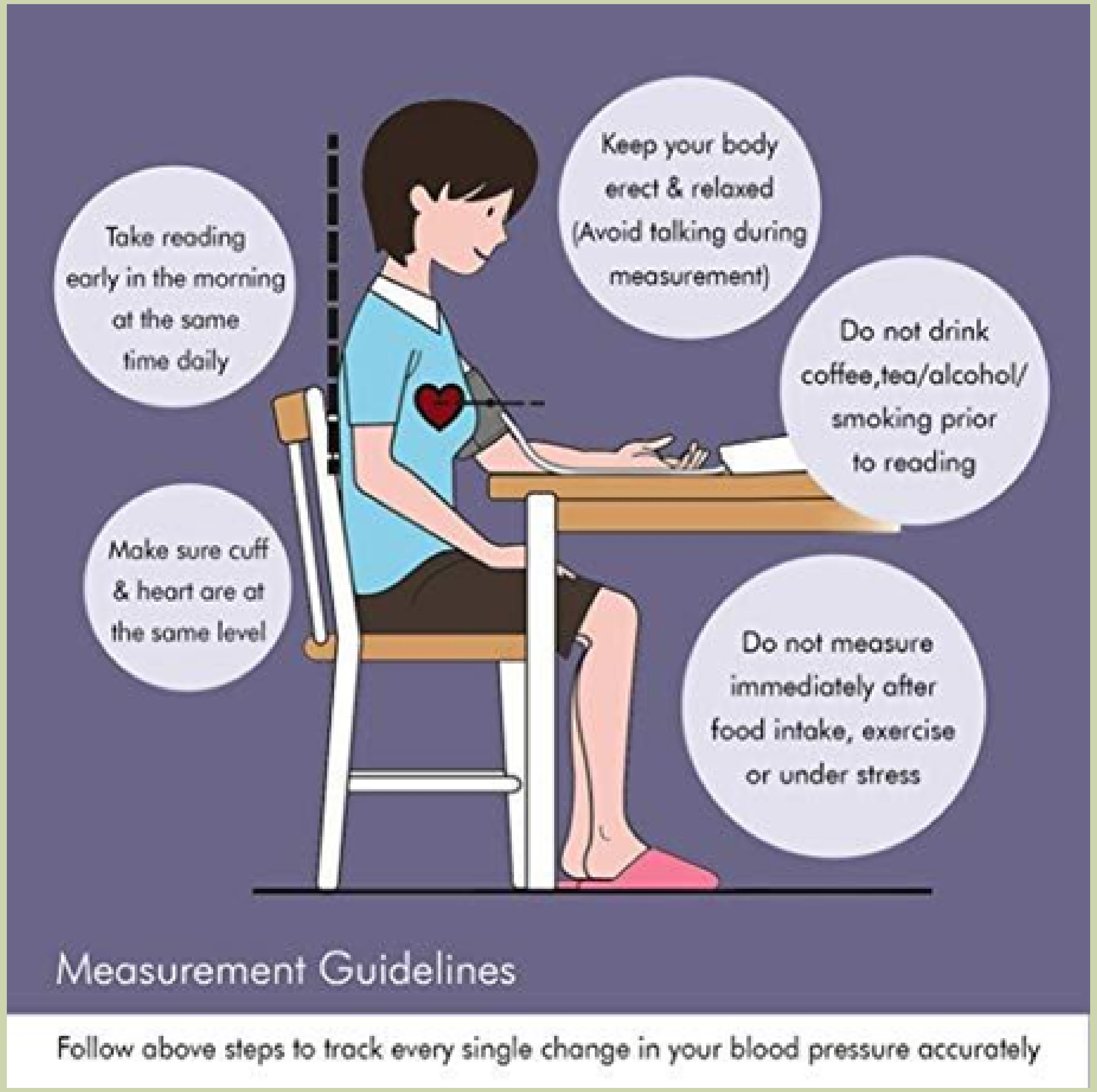

- Measure in the **morning** with documentation (Values will be used for research use)

Hypertension

1. Measurement of blood pressure

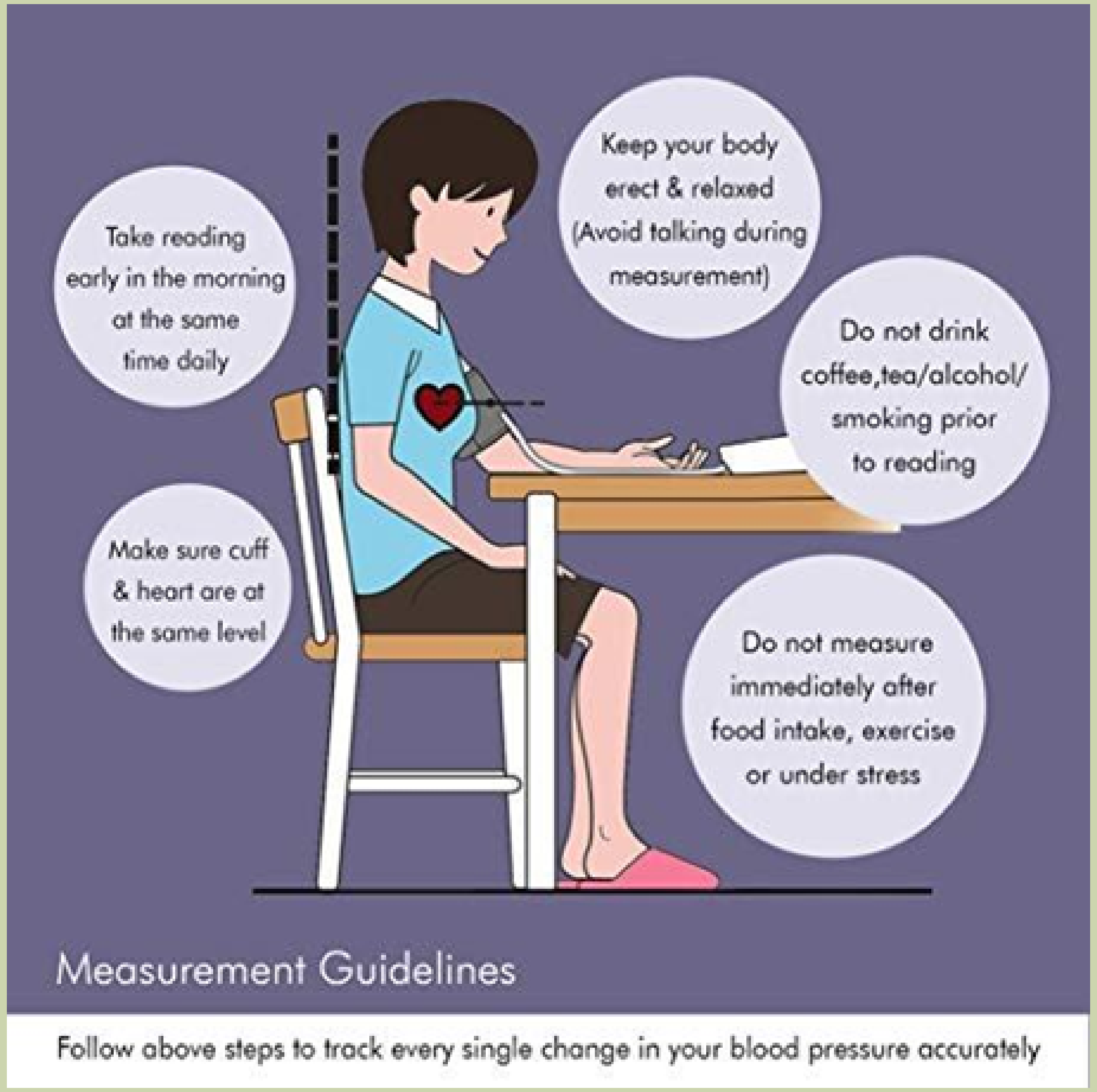

-**No talking** when measuring

-Drinking **coffee/tea/exercise** will affect the readings

- Better to **take a 15-min rest** before measurement

Hypertension

Medication

1. Diuretics -- Lasix
2. Calcium channel blocker -- Norvasc
3. Vasodilator -- Hydralazine
4. Alpha blocker -- Prazosin
5. Beta-blocker -- Betaloc
6. ACE inhibitor -- Lisinopril
7. Angiotensin II receptor blocker -- Losartan

Hypertension

Exercise prescription

Hypertension

Exercise prescription

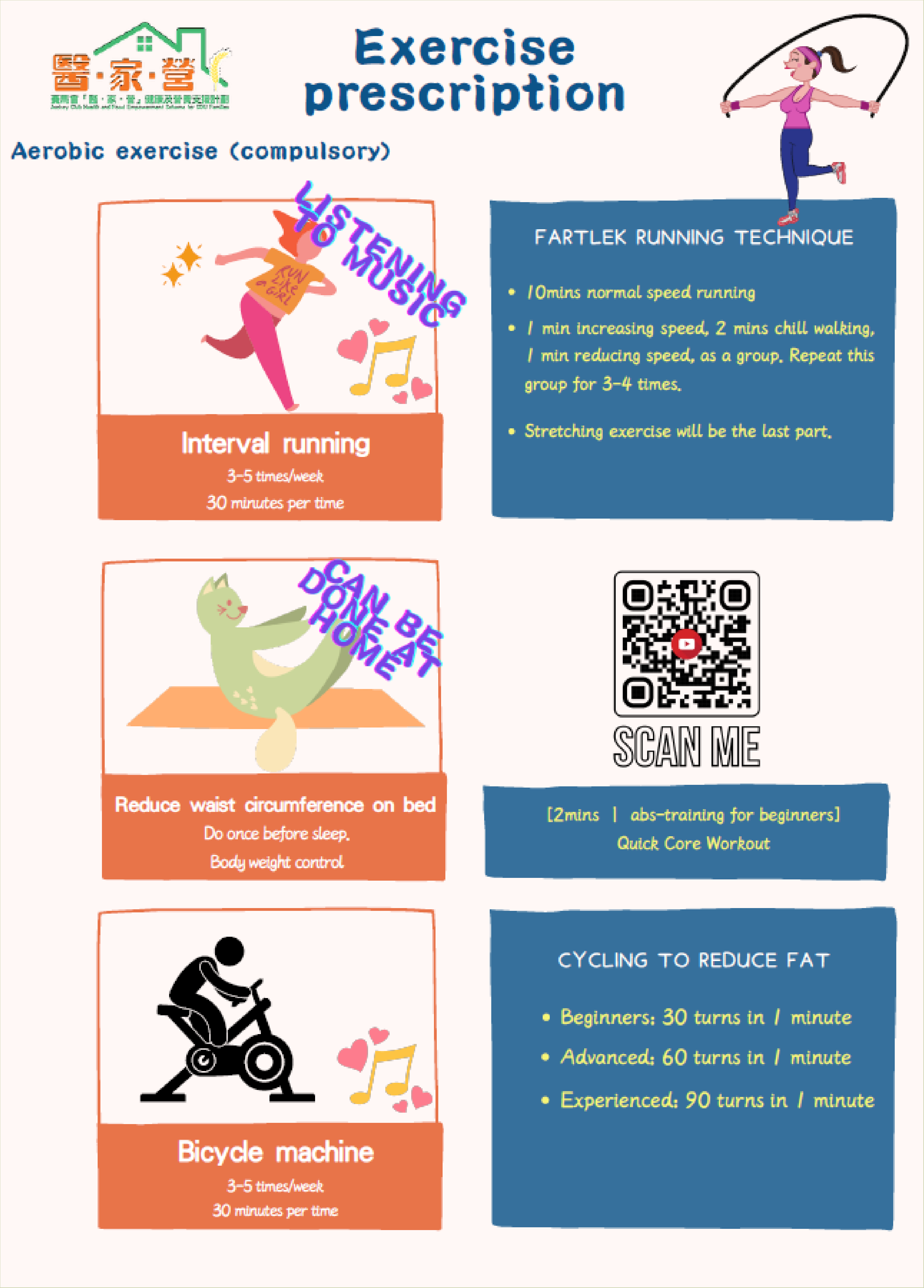

Hypertension

Exercise prescription

醫·家·營

BEGINNERS

Resistance exercise: 10-15 times as a group, perform 3-4 groups

Cushion Core exercise: glute-bridge

Alert
- Keep Leg still
- Maintain for 5 sec when your lower back reaches the highest point

Cushion Core exercise: Lower back training

Alert
- Keep hand straight
- Maintain for 5 sec when your lower back reaches the highest point

Cushion Core exercise: Abs-training

Alert
- keep legs at 90 degree
- Stick the back well to the floor

Umbrella Core exercise: Rotation

Alert
- Ensure enough space for doing this exercise
- Can be done in standing mode

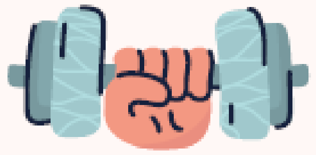

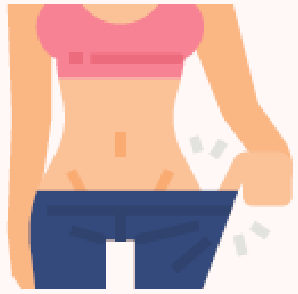

Hypertension

Exercise prescription

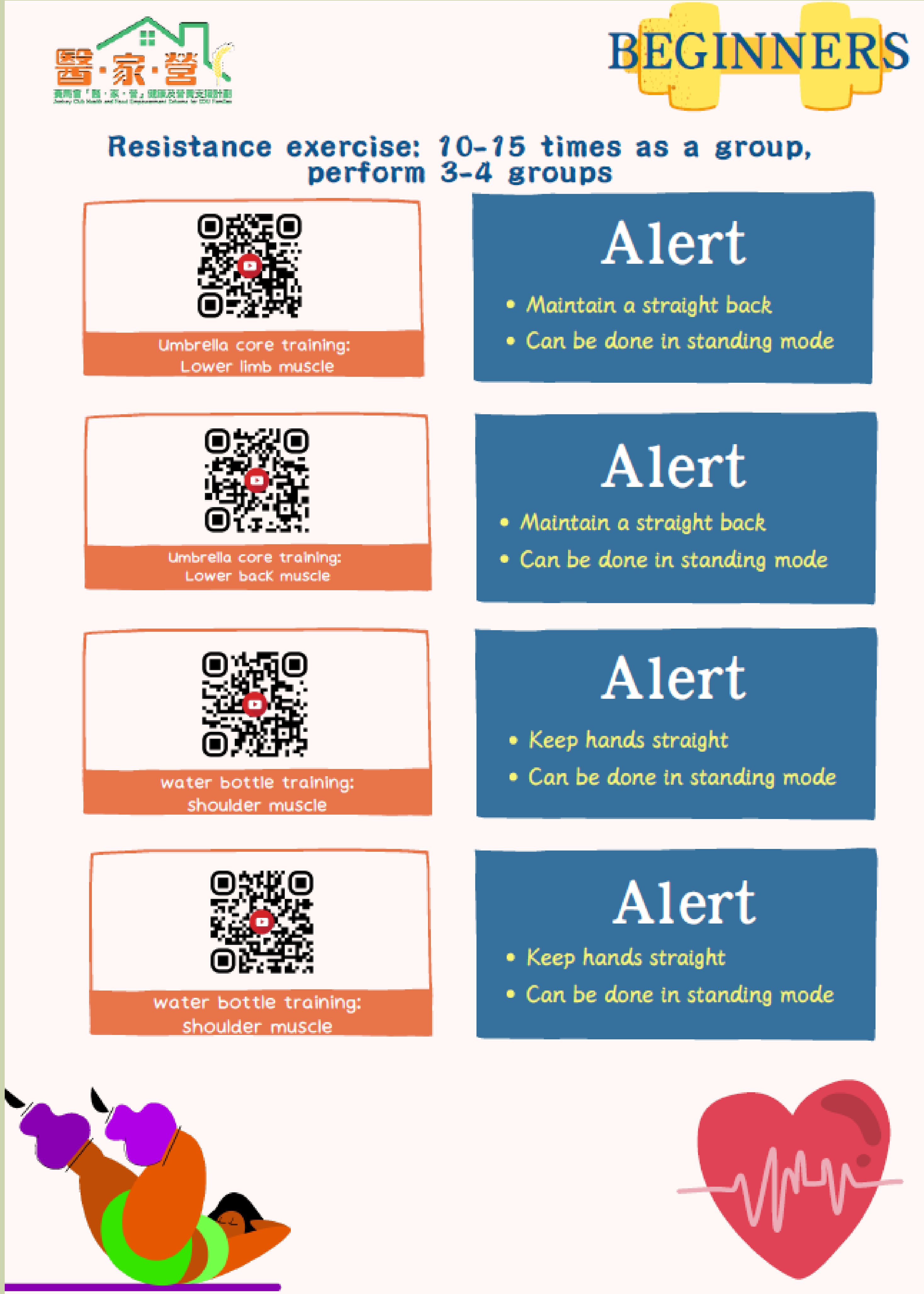

Hypertension

Exercise prescription

Advanced

Resistance exercise: 10-15 times as a group, perform 3-4 groups

water bottle training:
Biceps muscle

Alert

- No need to keep hand straight
- Can be done in standing mode

water bottle training:
Back core muscle

Alert

- Can be done in sitting mode

water bottle training:
Back core muscle

Alert

- Train back muscles and triceps

water bottle training:
Lower limb muscle

Alert

- Keep back straight and leg curved

Hypertension

Exercise prescription

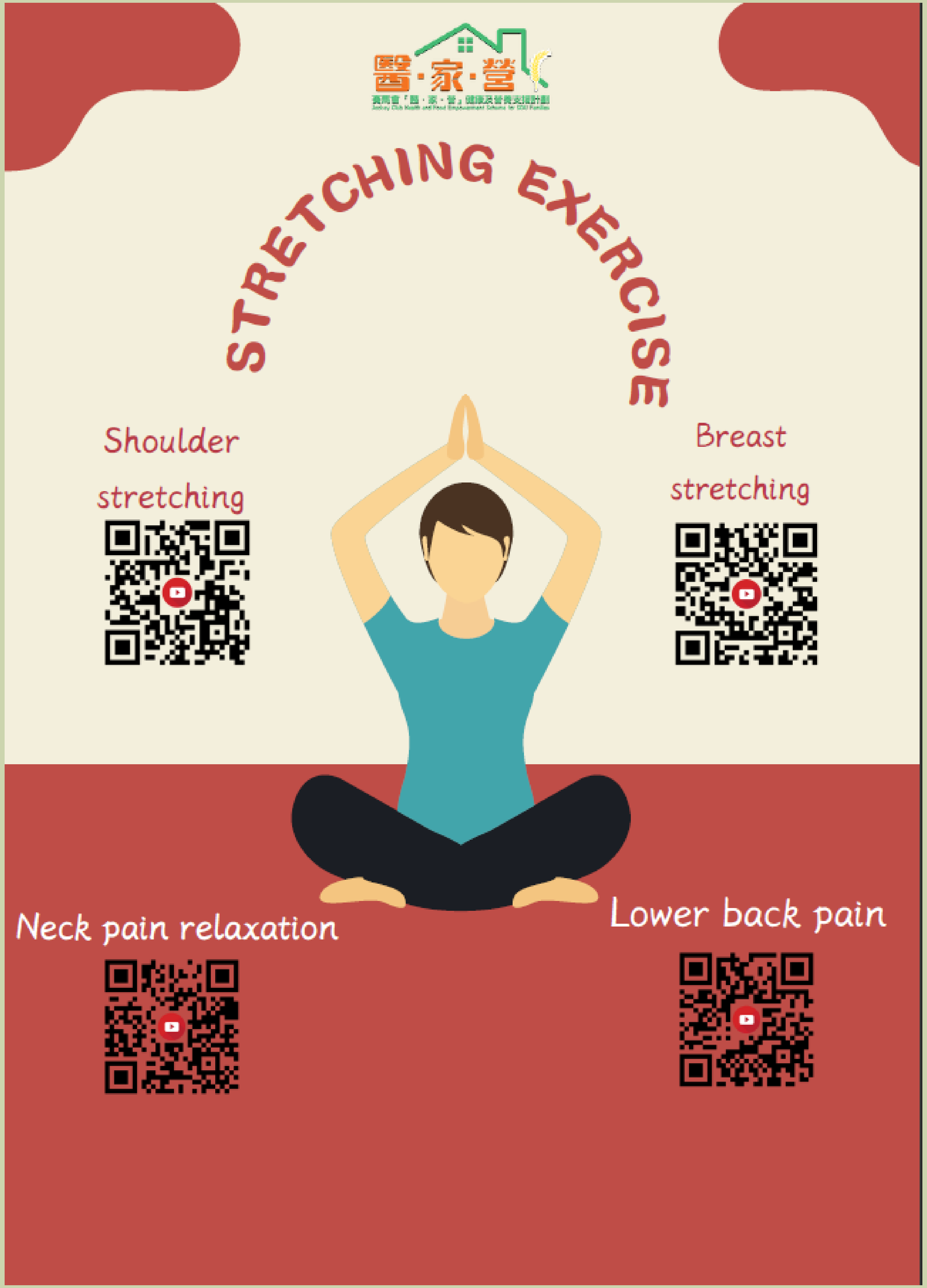

Hypertension

Case management (important questions to be asked)

1. Client's background (Past medical history)
2. Current goals given to client -- mainly on DASH Diet
3. Current range of blood pressure reading
4. Any drug allergy
5. Medication taking
6. Compliance of medication
    a. Right Dosage?
    b. Right Frequency?
    c. Right Drug?
    d. Right route?
    e. Right Patient?

Hypertension

Case management (important questions to be asked)

1. Mark down notes on progress notes (e.g BP reading, compliance on goals good/fair/poor, Next FU date and time)
2. Any referral to Nurse/Dietitian/SW?? (difficult problems which you feel like you can't manage well)

Hypertension

Case management (important questions to be asked)

1. Mark down notes on progress notes (e.g BP reading, compliance on goals good/fair/poor, Next FU date and time)
2. Any referral to Nurse/Dietitian/SW?? (difficult problems which you feel like you can't manage well)
3. Application of **stages of change model**

Hypertension

Case management (important questions to be asked)

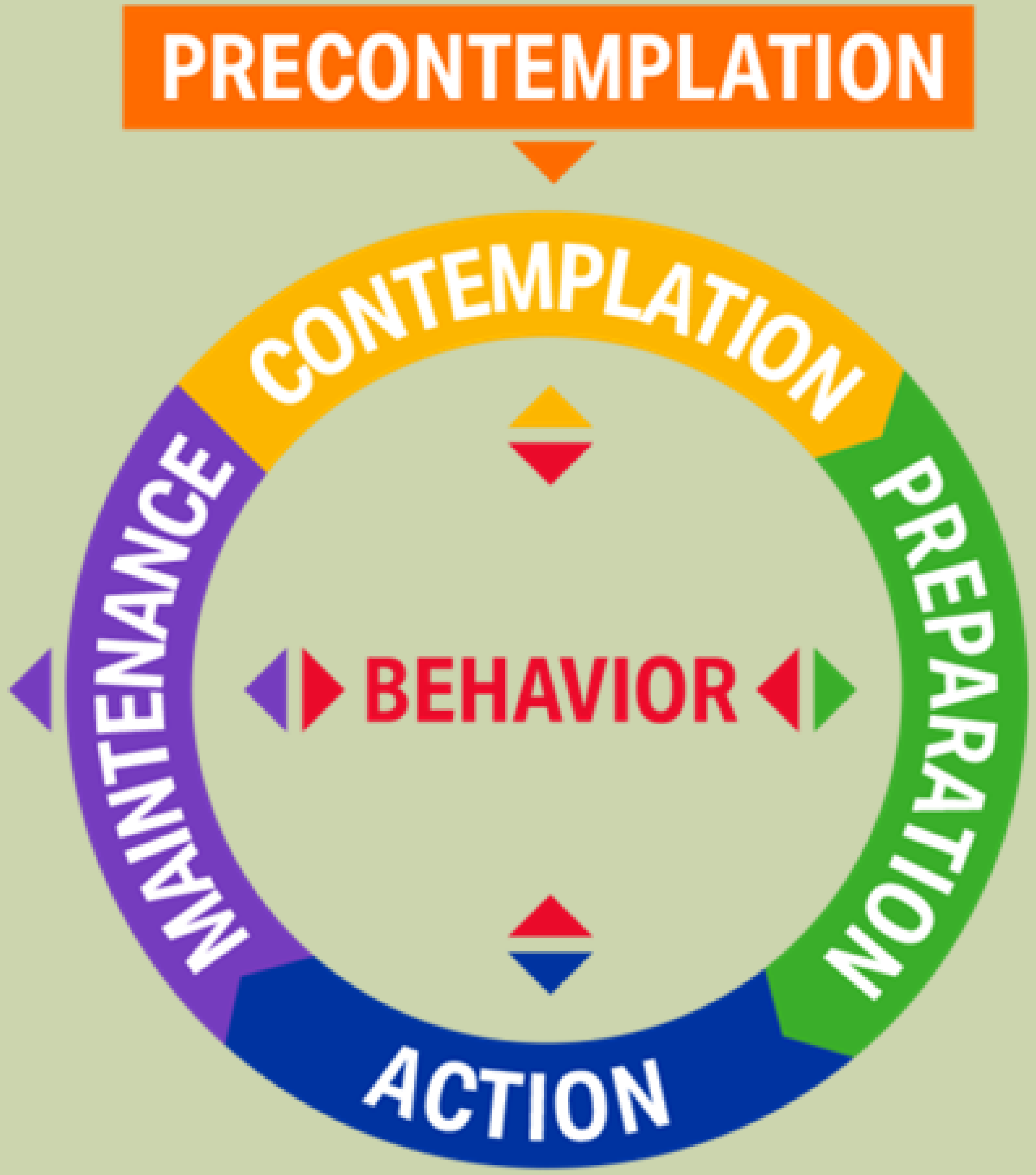

PRECONTEMPLATION
Build awareness for my need to change

CONTEMPLATION
Increase my pros for change and decrease my cons

PREPARATION
Commit and plan

ACTION
Implement and revise my plan

MAINTENANCE
Integrate change into my lifestyle

Hypertension

Case management FAQ

1. I know I have to take Blood pressure medication. I don't have to know the drug name and the dosage!
ANS: You have to know the Blood pressure medication drug name and dosage in order to avoid mistakes in the frequency of taking meds.

People say taking blood pressure medication will damage our kidney. I am afraid of taking the meds.
ANS: Under a normal circumstance, BP meds won't damage our kidney. Yet, a poor control over blood pressure will lead to renal failure which require renal dialysis.

I got a good control over blood pressure. I stop the medication on my own.
ANS: Under a normal circumstance, you shouldn't stop the medication on your own. Please discuss with your doctor if the systolic blood pressure is kept at range of 110mmHg to 120 mmHg.

My friends got hypertension too. I wanna share the meds with my friends.
ANS: Different people have different body condition. Please advise for doctor consultation if your friends got hypertension!

Diabetes Mellitus

1. Definition:
    a. HbA1c > 6.5% -- **golden rule**
    b. Fasting blood glucose > 6 mmol/L and Postprandial blood glucose > 11.1 mmol/L

Diabetes Mellitus

1. Type 1 DM:

-- autoimmune malfunction in releasing insulin

2. Type 2 DM

-- poor diet control, obesity

Diabetes Mellitus

Different type of Insulin

1. (Very short acting: Novorapid)
2. (Short acting: Actrapid)
3. (Middle acting: Humulin)
4. (Long acting: Lantus)

Diabetes Mellitus

Different type of Insulin

1. (Very short acting: Novorapid)
    a. Inject 15 mins before meal
    b. Please eat immediately after injection
2. (Short acting: Actrapid)
    a. Inject 30 mins before meal
    b. Please eat immediately after injection
3. (Middle acting: Humulin)
    a. Inject regularly every day
4. (Long acting: Lantus)
    a. Inject regularly every day

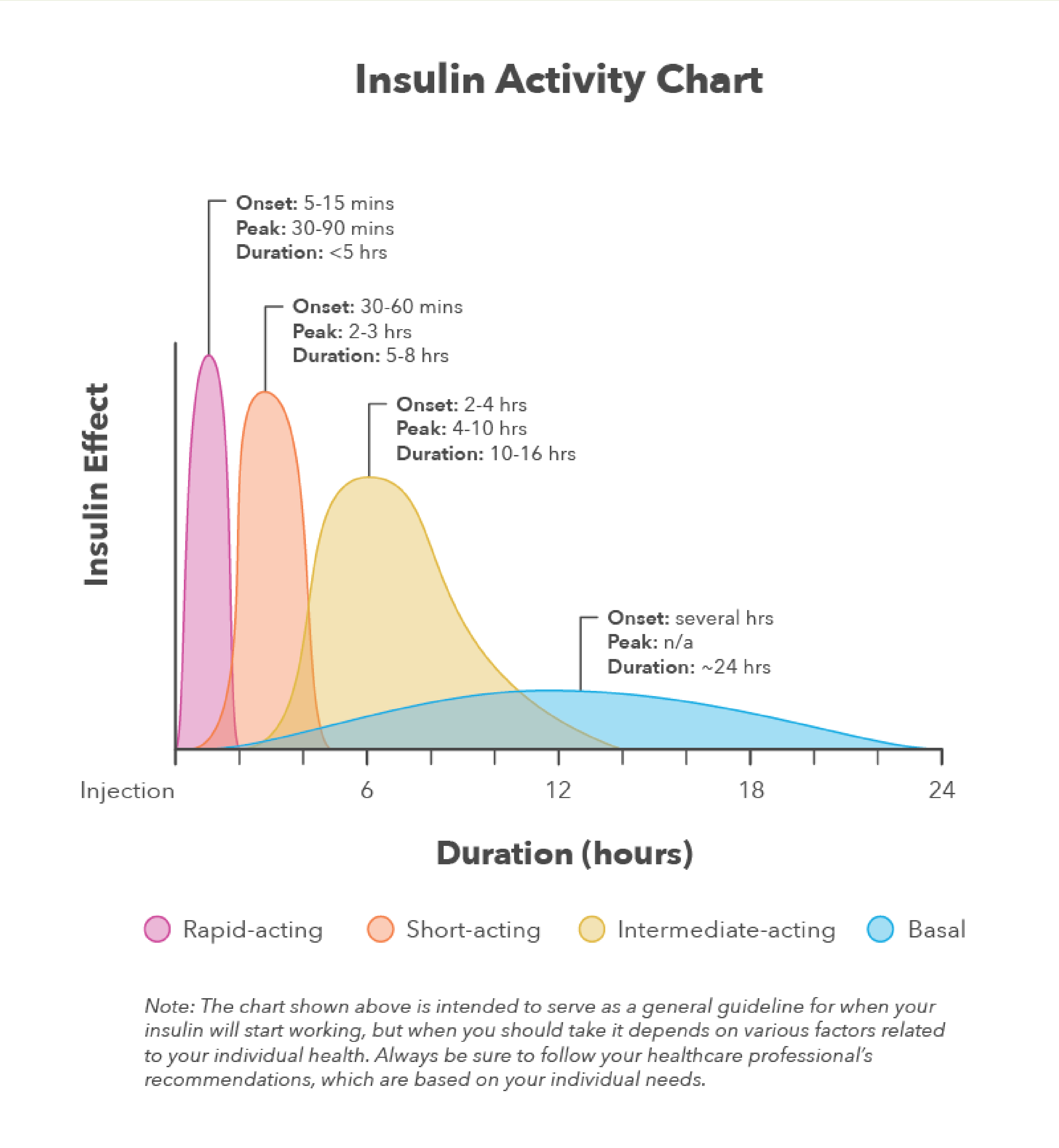

Diabetes Mellitus

Insulin application

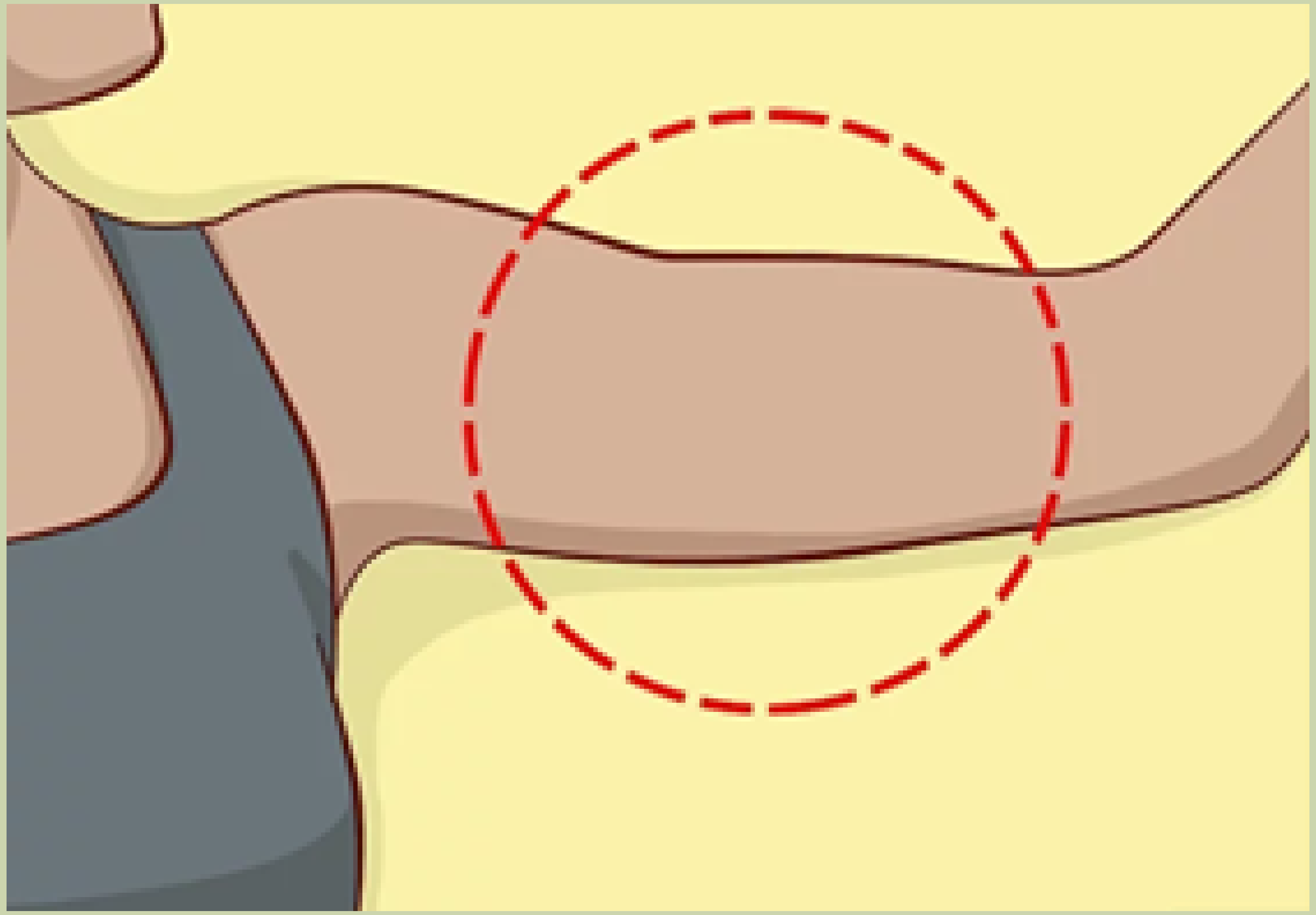

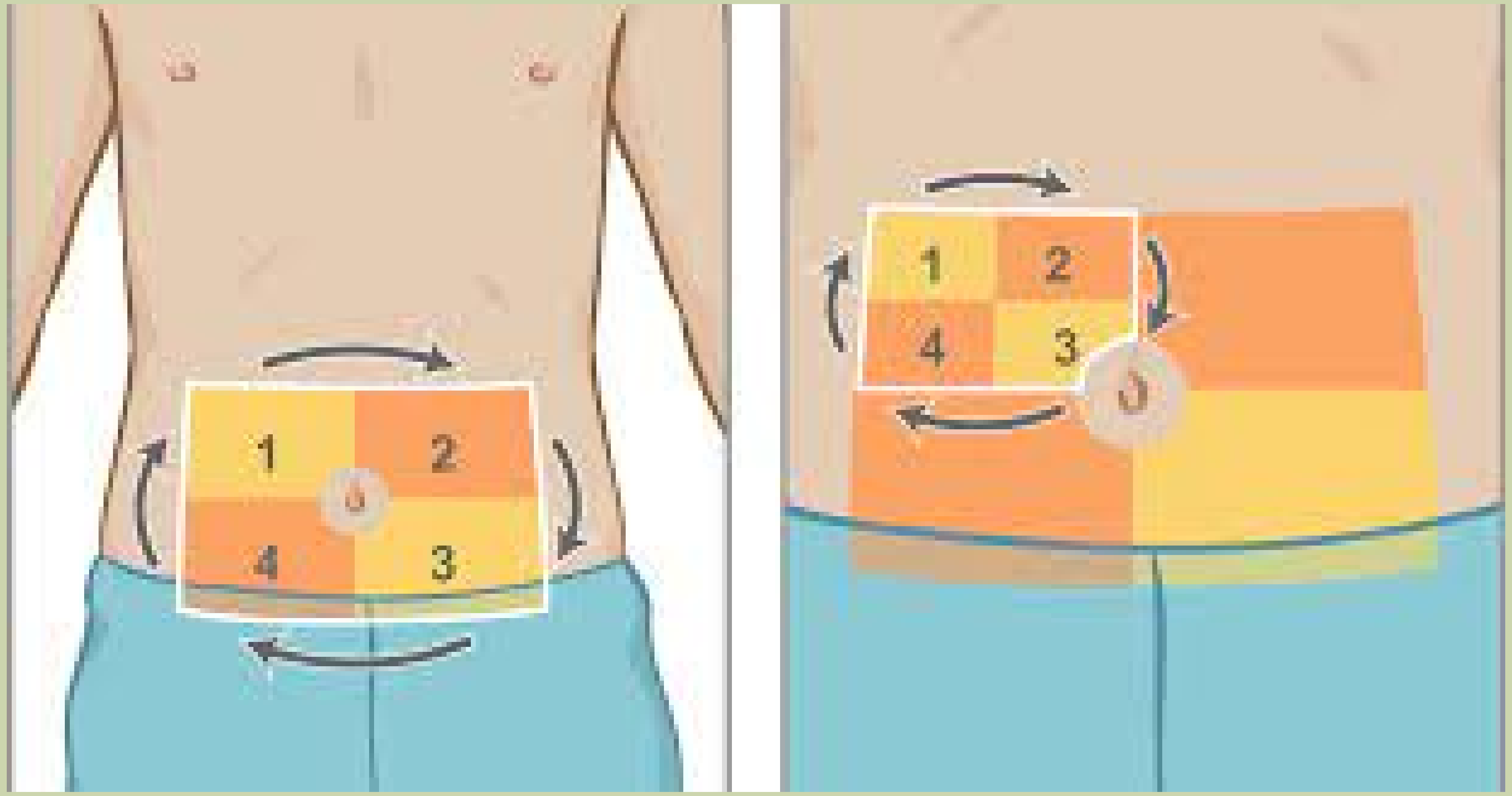

Diabetes Mellitus

Different type of oral medication

1. **(Sulphonylurea--Gliclazide, Glimepiride, Glipizide)**
2. **(Biguanides--Metformin)**
3. **(α-glucosidase inhibitor-Acarbose)**
4. **(Insulin sensitizer)**
5. (**Prandial glucose regulator)**
6. **(DPP-IV inhibitor—Vildagliptin, Linagliptin)**
7. **(Sodium-Glucose Co-transporter 2 Inhibitors—Canagliflozin, Dapagliflozin, Empagliflozin)**

Diabetes Mellitus

Forgot to take medication

Use your smart phone to set alarm!

If you forget to take med on time, please immediately take for missed dosage. Take the next dosage at assigned time!

Please remember not to take the med in over-dose.

Diabetes Mellitus

Case management (important questions to be asked)

1. Client's background (Past medical history)
2. Current goals given to client -- mainly on sugar reducing Diet, learn to calculate carbohydrate exchange.
3. Current range of blood glucose reading
4. Any drug allergy
5. Medication taking
6. Compliance of medication
    a. Right Dosage?
    b. Right Frequency?
    c. Right Drug?
    d. Right route?
    e. Right Patient?

Diabetes Mellitus

Exercise prescription

Diabetes Mellitus

Risk management

1. DKA (Diabetic Ketoacidosis) (due to forgetting insulin injection/med)
    a. Test for urine pH if Hstix >15mmol/L
    b. Go to A&E if Hstix > 25 mmol/L
2. HHS (Hyperglycemic hyperosmolar syndrome)

Diabetes Mellitus

Risk management

1. Remind client to eat equally and timely
2. Maintain good medication compliance

Diabetes Mellitus

Risk management

1. Hypoglycemia (Hstix < 4 mmol/L)
2. Symptoms: Chills and rigor, palpitation, dizziness, fatique, agitated.

Diabetes Mellitus

Risk management

1. Hypoglycemia (Hstix < 4 mmol/L)

- **Immediately drink one pack of white sugar mixed with water**
- Test for blood glucose after 15 mins.
- If your blood glucose is still below 4 mmol/L, you should drink sugar water again.
-Please go to A&E if your blood sugar can't rise after 3 sugar water trial

Pre-Diabetes Mellitus

1. Definition:
    a. HbA1c > 5.7% -- **golden rule**
2. Most of the cases will be in **pre-DM state**
3. We will follow the route based on **Diet changes, exercise prescription and blood glucose monitoring**

Pre-Diabetes Mellitus

1. Definition:
    a. HbA1c > 5.7% -- **golden rule**
2. Most of the cases will be in pre-DM state
3. We will follow the route based on **Diet changes and exercise prescription, blood glucose monitoring**

Pre-Diabetes Mellitus

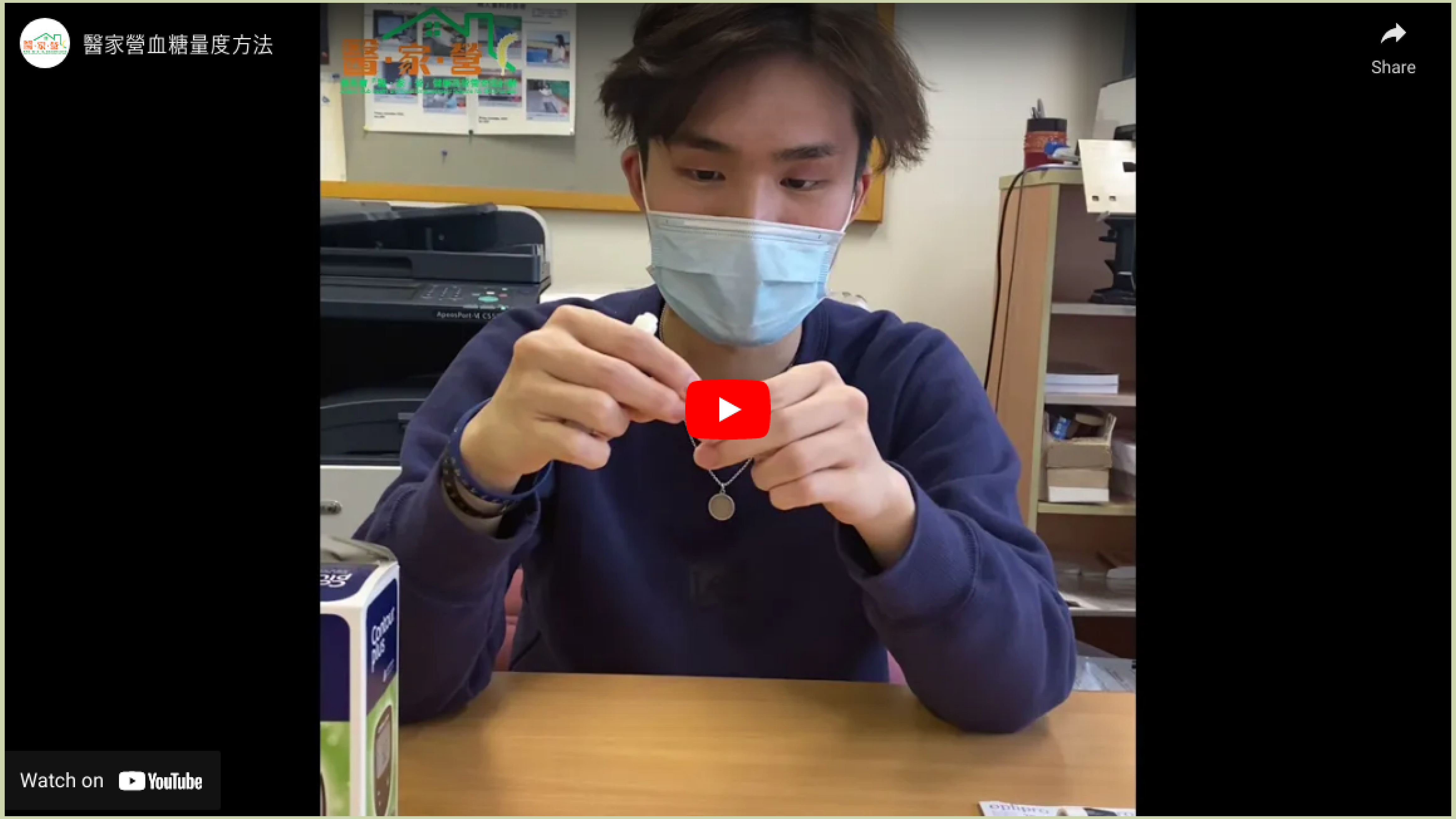

Pre-Diabetes Mellitus

Exercise prescription

Examples

Diet-Nur[illegible]

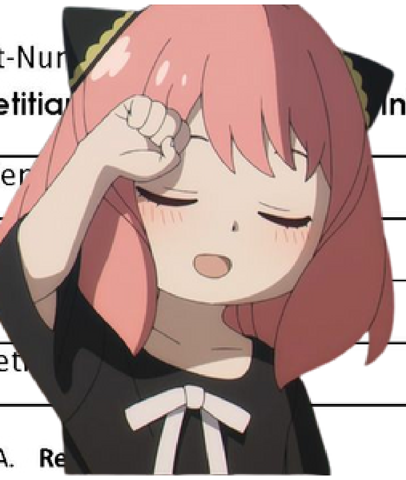

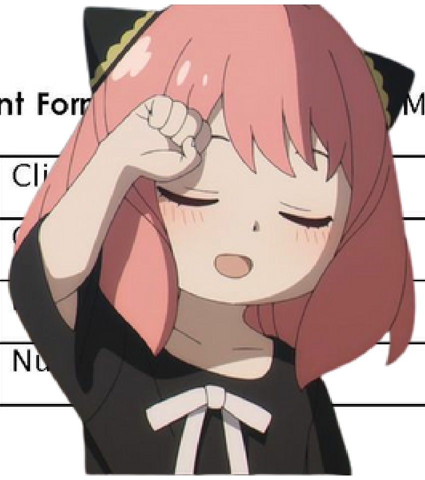

Dietitia[illegible] Initial Assessment For[illegible] May 26 2022

Clien[illegible]	Cli[illegible]
NC[illegible]	[illegible]
D[illegible]	[illegible]
Diet[illegible]	Nu[illegible]

A. **Re[illegible]**

❑ Weight management (BMI = 28.2)	❑ Suboptimal waist circumference (WC= cm)
❑ Abnormal Fasting Glucose (_)	❑ Abnormal Total Cholesterol (______________)
❑ Family history of ____________________	❑ Pre-Hypertension management
❑ Suboptimal dietary habits/ intakes e.g	❑ Pre-DM management (Hba1c: 6.0 %.)
❑ Poor Blood pressure control, Hypertension	❑ Poor BO, urine pattern
❑ Poor Drug compliance	❑ Poor DM Control
❑ Airway management	❑ Hypoxia
❑ Fall risk	❑ Risk of aspiration
❑ Family planning consultation	❑ Unknown bruise
	❑ Others: ______

B. Assessment summary

PES statement

1. Obesity related to insulin resistance or hormonal imbalance 2o to PCOS, impaired metabolism 2o to over-dieting and inadequate nutrition related knowledge as evidenced by BMI = 28.5, BW remained stable despite low energy intake

2. Inadequate protein and energy intake related to misconception on diet and weight loss as evidenced by complaints of easily fatigue and hair loss , DHx showed protein intake less than <2 serves/day and skipping Carbohydrates.

Chief C/O: Constipation

Long term goals:

1. 避免過度節食以改善身體新陳代謝
2. 六個月後慢慢減到76公斤
3. 糖化血紅素下降至5.7%以下

1

Examples

Diet-Nurse initial 2021

Short term goals

1. 跟隨健康飲食餐盤的飲食比例
2. 三餐正餐必須有碳水化合物、蛋白質及蔬菜
3. 早餐包含2份蛋白質食物; 午、晚餐包含2-3份蛋白質食物 (見蛋白質份量圖)
4. 每餐碳水化合物份量：三滿湯匙飯，6湯匙燕麥，1碗通粉，兩塊麵包，6隻雞蛋大小蕃薯
5. 用全穀物、豆類、燕麥、薯仔、蕃薯代替一半白米飯以增加纖維攝入量
6. 繼續現時蔬菜食量，但增加多元性，午餐和晚餐吃最少兩款蔬菜
7. 每天飲用脫脂奶1杯
8. 每周進食三次進食種子或豆類製品，如芝麻、南瓜子、豆乾、豆腐、枝豆，以改善荷爾蒙平衡
9. 每餐進食*一種*有助睡眠調節及減少脫髮的食物：牛奶、麥皮、黑芝麻、南瓜子、去皮雞肉、香蕉、豆類、豆腐、蕃薯、薯仔、雞蛋、三文魚、鯖魚、蝦仁、瘦肉
10. 每星期進行體重測試一次
11. 每星期五早上量度空腹血糖一次, 將數字紀錄在手機內 (LHW 請提問量度方法，於第四及第六次FU 前叫街坊量度空腹及餐後2小時的血糖)
12. 根據運動處方:
    a. 每星期做3-5 次間歇速步
    b. 1天選擇任何3款初階阻力運動, 10-15 下為一組, 做3-4組
    c. 每日晚上做一次伸展運動

Health recommendations

Other concerns (e.g________________________________)

- ❑ Require   to FU on problem:
- ❑ Nil concern

C. Patient background

Medical related:	Medical History:
1. Family history<br><br>Gout<br><br>2. Prescription<br><br>Nil	GDM (poor control)<br>PCOS (dx on may 26 on OBG visit; c/o irregular period)

Examples

Diet-Nurse initial 2021

Religion: Nil	Financial assistance: nil

Dietary related:

Dietary Limitation: nil

Drug Allergies & Food Sensitivities: nil.

Likes: nil

Dislikes: nil

Environmental (Economic and social status) living

Job status: Office work x5/7 (work when kids are in school)

Financial status:

Household member: 4 adult + 9y B + 5y G (clients + clients' dad)

Marital status: husband working thailand

Cooking facilities:

Food budget: $1-2/ week

Food assistance: yan chak sin food bank

Lifestyle:

Exercise: 1-2/ 7 x 健康操

Smoking: Nil

Alcohol: nil

Stages of change

Action – been on diet since last sep; very persistent and been following the same diet pattern for month

Motivation: easing her knee pain, prevent hair loss, and boost energy level

Examples

Diet-Nurse initial 2021

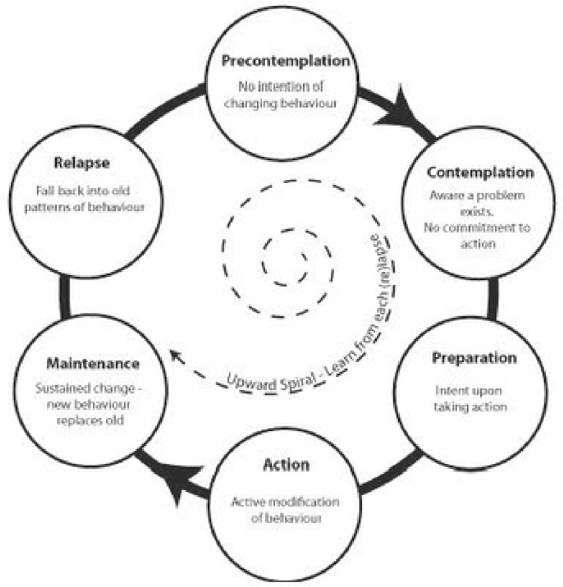

D. Assessment

Anthropometry Ht 158cm<br>1. Current body weight: (may 2022) 70.3kg, BMI 28.2, %BF 37.2<br>2. Body weight history: 75kg (09/2021) ; On diet 9/12 ago – **skipped CHO + Exercise (dance); lost 10lb to 68kg**
Biochemistry<br>BP: 121/76mmHg<br>Heart rate: 84 BPM<br>Hstix: 8.1<br>Hb: 12.2<br>Hct: 36%<br>HbA1c: **6.0%**
Clinical:<br>c/o knee pain related to long hours of standing (alleviated after quitting 711 job)<br>C/o hair loss, easily fatigue,<br>1. Eyesight: Normal<br>2. Hearing: Normal<br>3. Smelling: Normal<br>4. Chest Xray done within half year? Nil

Examples

5. **Pain? Nil**

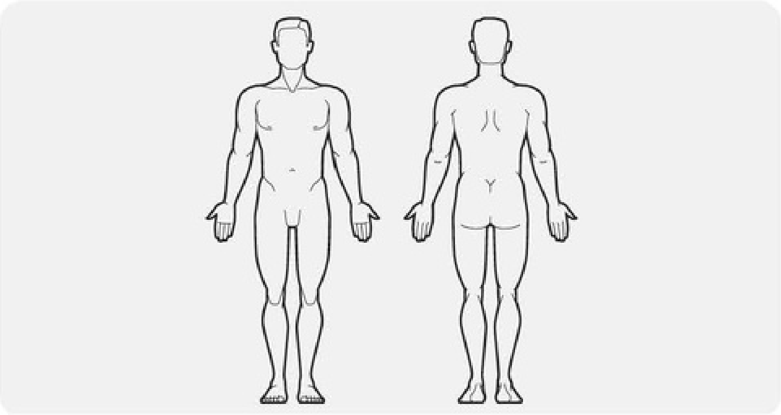

6. **Muscle strength: full**

7. **Skin integrity: intact, no suspicious bruising or delayed wound healing**

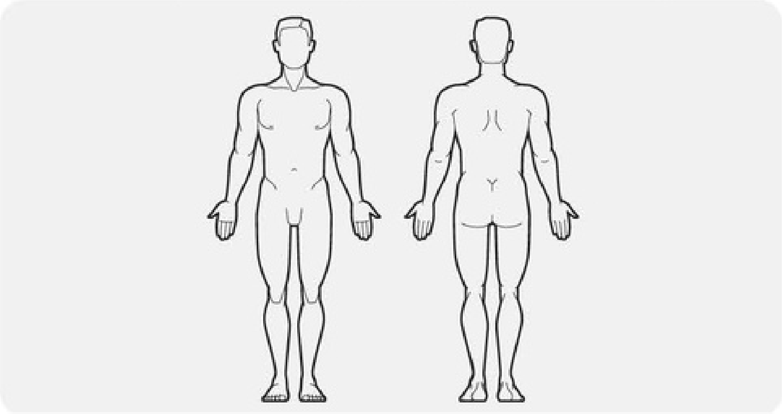

Diet

Inadequate protein, dairy, F&V

Environmental (Economic & Social Status) See Above

Education/ Electronic gadgets provided:

- **Written resource: general healthy eating 1.2, JChomes kitchen list, protein serve, overweight diet resource and exercise perscription**
- **Explained the correct concept of weight loss which is gaining muscle mass and losing fat mass , also mentioned harder to lose weight in view of PCOS + hormonal imbalance + insulin resistance**
- **Provided BGL monitor; scale not needed**

Plan:
1st FU phone June 9 4:30pm

Review:

The end

S5. Interview guide for process evaluation

#	Questions
A1	Why is the intervention being implemented in your setting? Who decided to implement the intervention? How was the decision made to implement the intervention?
A1	What do you know about the intervention or its implementation?
A1	Has the intervention been implemented according to the implementation plan?<br>o [If Yes] Can you describe this? o [If No] Why not?
A2	What kind of supporting evidence or proof is needed about the effectiveness of the intervention to get staff on board? Co-workers? Administrative leaders?
A2	How does the intervention compare to other alternatives that may have been considered or that you know about? What advantages does the intervention have compared to these other programs? What disadvantages does the intervention have compared to these other programs?
A2	Do you think the intervention will be effective in your setting? Why or why not?
A3	What barriers will the individuals served by your organization face to participating in the intervention?
A4	What kinds of changes or alterations do you think you will need to make to the intervention so it will work effectively in your setting? Do you think you will be able to make these changes? Why or why not?
A4	Who will decide (or what is the process for deciding) whether changes are needed to the intervention so that it works well in your setting? How will you know if it is appropriate to make any changes?
A4	Are there components that should not be altered? Which ones should not be altered?
A5	Will the intervention be piloted prior to full-scale implementation? What was the pilot look like?
A5	How complicated is the intervention? (duration, scope, intricacy and number of steps involved)

A6	How confident are you that you will be able to successfully implement the intervention? What gives you that level of confidence (or lack of confidence)?
A6	How prepared are you to use the intervention? (Knowledge - Presuasion - Decision - Implementation - Confirmation)
A7	How confident do you think your colleagues feel about implementing the intervention? What gives them that level of confidence (or lack of confidence)?
B1	Can you describe your working relationships with your colleagues? With colleagues in your unit? With colleagues in other units? Can you tell me a story about a time you needed to work with others to solve a problem? Or to implement an intervention in the past or this intervention?
B10	Will feedback be elicited from staff? From the individuals served by your organization? [If yes] What kind of feedback?
B2	Can you describe your working relationship with leaders? Your supervisor? Supervisors of other colleagues?
B3-B4	What level of involvement/ support has leadership at your organization had so far with the intervention? Do they know about the intention to implement the intervention? Who are these leaders? How do attitudes of different leaders vary? What kind of support have they given you ? Can you provide specific examples? What types of barriers might they create?
B5	When you need to get something done or to solve a problem, who are your "go-to" people? Can you describe a recent example?
B6	What are influential individuals saying about the intervention? Who are these influential individuals? To what extent will they influence others' use of the intervention? The success of the implementation?
B7	Other than the formal implementation leader, are there people in your organization who are likely to champion (go above and beyond what might be expected) the intervention? Were they formally appointed in this position, or was it an informal role? What position do these champions have in your organization? What kinds of behaviors or actions do you think this individual/champion will exhibit?
B8	Will someone (or a team) outside your organization be helping you with implementing the intervention? Can you describe this person/group? How did they get involved? What is their role? What kind of activities will they be doing? How helpful do you think he/she/they will be? In what ways?

B9	What steps have been taken to encourage individuals to commit to using the intervention? What is your communication or education strategy (not including training, see Access to Knowledge and Information) for getting the word out about the intervention?
B9	How will you or your colleagues communicate to the individuals that are served by your organization about the intervention? How will they participate in the intervention? How will they access the intervention?
C1	To what extent is staff aware of the needs and preferences of the individuals being served by your organization? How "in touch" are staff and leadership with the individuals served by your organization?
C1	To what extent were the needs and preferences of the individuals served by your organization considered when deciding to implement the intervention? Can you describe specific examples? Will the intervention be altered to meet their needs and preferences?
C1	How well do you think the intervention will meet the needs of the individuals served by your organization? In what ways will the intervention meet their needs? Eg improved access to services? Reduced wait times? Help with self-management? Reduced travel time and expense ? How do you think the individuals served by your organization will respond to the intervention?
C2	Do you expect to have sufficient resources to implement and administer the intervention? What resources are you counting on? Are there any other resources that you received, or would have liked to receive? What resources will be easy to procure? What resources will not be available?
C3	What kinds of information and materials about the intervention have already been made available to you? Copies of materials? Personal contact? Internal information sharing; eg, staff meetings? Has it been timely? Relevant? Sufficient ?
C4	Can you tell me what you know about any other organizations that have implemented the intervention or other similar programs? How has this information influenced the decision to implement the intervention?
C5	What kind of local, state, or national performance measures, policies, regulations, or guidelines influenced the decision to implement the intervention? How will the intervention affect your organization's ability to meet these measures, policies, regulations, or guidelines?

C6	How do you feel about the intervention being used in your setting? How do you feel about the plan to implement the intervention in your setting? Do you have any feelings of anticipation? Stress? Enthusiasm? Why?
D1	What kind of information exchange do you have with others outside your setting, either related to the intervention, or more generally about your profession? What professional networking do you engage in? Listservs? Local or national conferences? Trainings?
D1	To what extent does your organization encourage you to network with colleagues outside your own setting? Are you able to attend local/national conferences? Other venues?
D2	How will the infrastructure of your organization (social architecture, age, maturity, size, or physical layout) affect the implementation of the intervention? How will the infrastructure facilitate/hinder implementation of the intervention? How will you work around structural challenges?
D2	What kinds of infrastructure changes will be needed to accommodate the intervention? Changes in scope of practice? Changes in formal policies? Changes in information systems or electronic records systems? Other? What kind of approvals will be needed? Who will need to be involved? Can you describe the process that will be needed to make these changes?
D3	How would you describe the culture of your organization? Of your own setting or unit? Do you feel like the culture of your own unit is different from the overall organization? In what ways?
D3	How do you think your organization's culture (general beliefs, values, assumptions that people embrace) will affect the implementation of the intervention? Can you describe an example that highlights this?
D7	What is the general level of receptivity in your organization to implement the intervention? Why?
E2	What have you done (or what do you plan to do) to get a plan in place to implement the intervention? What is your role in the planning process? Who is involved in the planning process? What are their roles? Are the appropriate people involved in the planning process? How engaged are they?
E3	How will you assess progress towards implementation or intervention goals?<br>o How will results of the evaluation be distributed to stakeholders?

E4	To what extent has your organization/unit set goals for implementing the intervention? How will goals be communicated in the organization? To whom will they be communicated? What are the goals? How and to whom will they be communicated?
